# Bioactive Compounds with Pesticide Activities Derived from Aged Cultures of Green Microalgae

**DOI:** 10.3390/biology12081149

**Published:** 2023-08-19

**Authors:** Alethia A. Brito-Bello, Damar Lopez-Arredondo

**Affiliations:** Institute of Genomics for Crop Abiotic Stress Tolerance, Department of Plant and Soil Science, Texas Tech University, Lubbock, TX 79409, USA

**Keywords:** green microalgae, allelochemicals, biopesticides, Chlorophyta, nontargeted metabolomics

## Abstract

**Simple Summary:**

Overusing synthetic pesticides has resulted in environmental problems and human health risks while contributing to pesticide-resistant pests. Microalgae, being natural producers of highly diverse bioactive compounds, represent a promising alternative to replace synthetic pesticides as a more environmentally friendly and safe alternative. In this study, we screened biomass extracts from 10 green microalgae from the Chlorophyta phylum to identify molecules with potential pesticide activities. All tested strains exhibited herbicidal, nematocidal, or algicidal activities. Metabolomics analysis of six strains showed the presence of fatty acids, isoquinoline alkaloids, and aldehydes, which may be responsible for the observed pesticide activities. Our study highlights the algicidal, herbicidal, and nematocidal activities of biomass extracts derived from green microalgae and the metabolites and metabolic pathways potentially involved in producing the bioactive molecules. Our study supports the potential of microalgae for developing more effective and eco-friendlier biopesticides for sustainable agriculture.

**Abstract:**

The excessive use of synthetic pesticides has caused environmental problems and human health risks and increased the development of resistance in several organisms. Allelochemicals, secondary metabolites produced as part of the defense mechanisms in plants and microorganisms, are an attractive alternative to replace synthetic pesticides to remediate these problems. Microalgae are natural producers of a wide range of allelochemicals. Thus, they provide new opportunities to identify secondary metabolites with pesticide activities and an alternative approach to discover new modes of action and circumvent resistance. We screened 10 green microalgae strains belonging to the Chlorophyta phylum for their potential to inhibit the growth of photosynthetic and nonphotosynthetic organisms. Bioassays were established to assess microalgae extracts’ effectiveness in controlling the growth of *Chlorella sorokiniana*, *Arabidopsis thaliana*, *Amaranthus palmeri*, and the model nematode *Caenorhabditis elegans*. All tested strains exhibited herbicidal, nematocidal, or algicidal activities. Importantly, methanol extracts of a *Chlamydomonas* strain effectively controlled the germination and growth of a glyphosate-resistant *A. palmeri* biotype. Likewise, some microalgae extracts effectively killed *C. elegans* L1 larvae. Comprehensive metabolic profiling using LC-MS of extracts with pesticide activities showed that the metabolite composition of *Chlamydomonas*, *Chlorella*, and *Chloroidium* extracts is diverse. Molecules such as fatty acids, isoquinoline alkaloids, aldehydes, and cinnamic acids were more abundant, suggesting their participation in the pesticide activities.

## 1. Introduction 

Synthetic pesticides in agriculture have reached record levels globally, with approximately 5.2 billion pounds being utilized annually worldwide [1]. Synthetic pesticides have proven effective in controlling pests and enhancing food production. However, their extensive use has resulted in several detrimental consequences. Pests have developed resistance to various pesticide classes, thus, imposing significant challenges to building sustainable agricultural practices for current and future crop cultivation [2]. Furthermore, these chemicals have been associated with environmental damage, harm to nontarget organisms, and negative impacts on human health [1,3]. For instance, more than 500 unique cases of herbicide-resistant weed biotypes (species/site of action) have been reported in more than 70 countries, affecting the cultivation of almost 100 crops [4]. In the U.S., 127 unique cases of herbicide-resistant weeds have been reported, primarily affecting wheat, corn, rice, soybean, and cotton cultivation [4]. These factors have led to an urgent need to develop new bioactive compounds with herbicidal activity and novel modes of action, safer toxicological and environmental profiles, and that are more efficient and selective than commercially available ones.

To address these concerns, exploring natural products as alternatives to synthetic pesticides in agriculture has gained traction [5,6,7,8,9,10]. Natural compounds offer several advantages, including their high biodegradability and low residuality, making them environmentally safe. Additionally, these compounds exhibit specificity toward target organisms, minimizing harm to nontarget species. Furthermore, they may have unique mechanisms of action, making it less likely for pests to develop resistance [5].

Microorganisms and plants have been found to produce a diverse array of natural compounds. Among these organisms, microalgae, unicellular photosynthetic organisms, stand out as prolific producers of biologically active metabolites. A rough estimate suggests that nearly 19,000 new compounds were identified and isolated from these sources in 40 years (from 1965 to 2006) [11]. However, many more remain undiscovered, requiring a concerted effort to discover and characterize new compounds in algae. This vast diversity of bioactive compounds might have evolved as part of the allelopathic capacity of microalgae [5]. Algal allelochemicals include diverse chemical classes such as phenolic compounds, terpenoids, free fatty acids, polysaccharides, alkaloids, and carotenoids with potential agricultural applications [12]. Previous studies on allelopathic activity in unicellular organisms have primarily focused on Cyanobacteria, photosynthetic bacteria that are often considered algae. Cyanobacteria are known for producing potent toxins called cyanotoxins that, unfortunately, have been found to have adverse effects on mammalian cells [13,14,15,16,17]. Further exploration is required to uncover new biomolecules of interest from different phyla of microalgae, such as Chlorophyta, because these algae generally have faster growth rates than Cyanobacteria and are easier to culture in mass [15]. 

Allelochemicals operate in different ways, i.e., affecting the growth of other algae, higher plants, or other microorganisms; they can be secreted or accumulated by the algae and cause growth inhibition or, in most cases, cell death [11,18,19,20,21]. Factors such as pH, light, and temperature changes have been identified as important triggers for producing allelochemicals in microalgae [11]. Additionally, nutrient limitation has been observed to enhance the production of allelochemicals in several green microalgae, either due to a lack of specific nutrients or prolonged cultivation in the same medium (aged cultures) [22]. These modes of activating allelochemical production were reported during the characterization of extracts for toxicity against other microalgae. For instance, aged cultures of *Chlorella vulgaris*, *Chlamydomonas reinhardtii*, and *Botryococcus braunii* exhibited algicidal activity attributed to a mixture of fatty acids. Similarly, growth-inhibiting substances were detected in aged cultures of members of the *Volvocaceae* family [23,24,25,26]. 

The algicidal activity of microalgae extracts has been widely studied, mainly from an ecological standpoint. However, their potential efficacy as herbicides or nematicides to control weeds and phytopathogenic nematodes still needs to be explored. Consequently, in this study, we investigated the allelopathic potential of aged cultures of green microalgae strains by conducting bioassays to evaluate their algicidal, nematocidal, and herbicidal activities. Chlorophyta strains from the *Chlorella*, *Chlamydomonas*, and *Chloroidium* genera were found to produce growth-inhibiting substances. Notably, the biomass extract of strain *C. reindhartii* CC124 effectively controlled the growth and germination of a glyphosate-resistant *A. palmeri* biotype, suggesting its potential as a bioherbicide. To gain insights into the chemical nature of metabolites potentially involved in these activities, metabolite profiling was performed using liquid chromatography-mass spectrometry (LC-MS) of microalgae extracts. Metabolite profiles were diverse with up to 45 different chemical classes of molecules. A metabolic pathway enrichment analysis suggests that more abundant metabolites in aged microalgae cultures are intermediates in different pathways, including betalain and isoquinoline alkaloid biosynthetic pathways. Numerous molecules produced through these pathways have been previously reported to have diverse bioactivities, suggesting that utilizing green microalgae as sources of bioactive compounds to develop sustainable agriculture practices holds great potential.

## 2. Materials and Methods

### 2.1. Microalgae Strains and Culture Conditions

*C. reinhardtii* strains CC125, CC124, LMJ.RY0402.054764, CC3054, and CC4533, hereafter strains 125, 124, 4764, 3054, and 4533, respectively, were obtained from the Chlamydomonas Resource Center, whereas *Chlorella* strains *Chlorella sorokiniana* UTEX 3016, *Chlorella* sp. UTEX EE162, *Chlorella* sp. ‘anitrata’ (formerly *Chlorella anitrata*) UTEX 1798, *Chlorella sorokiniana* UTEX 1230, *Chlorella sorokiniana* UTEX 1602, and *Chloroidium saccharophilum* (formerly *Chlorella saccharophila* var. *saccharophila*) UTEX 247, hereafter strains 3016, 162, 1798, 1230, 1602, and 247, respectively, were obtained from the UTEX Culture Collection of Algae of The University of Texas at Austin, USA. The axenicity of all strains was verified, and non-axenic ones were subjected to antibiotic treatment, washing by filtration, and isolation of single colonies. All microalgae were routinely cultivated under mixotrophic axenic conditions at 50 µmol m^−2^ s^−1^ constant illumination and 28 °C in a Conviron growth chamber model A1000 (Controlled Environments Inc., Pembina, ND, USA). Cells were cultivated in 50 mL glass flasks, 25 mL Tris-acetate-phosphate (TAP) [27] growth medium working volume, and constant shaking at 120–130 rpm. Bacto™ Agar (BD Difco, DF0479-17-3, Fisher Scientific, Waltham, MA, USA), 15 g/L, was added to the TAP medium for agar plates.

To search for algicidal, herbicidal, and nematocidal activities, batch cultures of each microalga were generated using homemade 1-L bioreactors with 900 mL of TAP medium working volume for 30 days. Cultures were grown under a 16:8 light/dark photoperiod, 50 µmol m^−2^ s^−1^ light intensity at 28 °C, and constant air bubbling in a Conviron growth chamber model A1000 (Controlled Environments Inc., Pembina, ND, USA). Batch cultures were inoculated with 1% of densely grown precultures to an OD_750_ of 0.2.

### 2.2. C. elegans Maintenance and Culture Conditions

*C. elegans* N2 (wild-type) and *Escherichia coli* OP50 were obtained from the Caenorhabditis Genetics Center at the University of Minnesota, USA. As reported before, *C. elegans* was maintained in nematode growth medium (NGM) agar plates at 20 °C and incubated in a Conviron growth chamber model A1000 (Controlled Environments Inc., Pembina, ND, USA) [28]. The worm was seeded with living *E. coli* OP50 as a food source. 

A life span synchronous assay was performed to assess the power of algal extracts to control the growth of *C. elegans.* As previously described, age-synchronized populations of *C. elegans* were obtained by hypochlorite treatment [29]. Briefly, worms grown on NGM agar were washed with sterile water and treated with 5 M NaOH and 5% NaOCl (1:2) for 8–10 min for nematode lysis. The lysate was pelleted by centrifugation (1200 rpm, 2 min) to obtain eggs. Then, the pellets were washed with sterile water at a 1:2 ratio, followed by centrifugation (1200 rpm, 2 min). Finally, the upper layer of water was removed, the egg pellet was collected, and eggs were allowed to hatch in the M9 buffer [29]. *C. elegans* L1 larvae were used for bioassays, as explained below.

### 2.3. Arabidopsis thaliana and Amaranthus palmeri Germination and Seed Multiplication

In this study, we used *A. thaliana* Col-0 ecotype and a glyphosate-resistant *A. palmeri* S. Wats. biotype, also known as Palmer amaranth or Palmer pigweed. The glyphosate-resistant *A. palmeri* biotype was purchased from the company Azlin seed service (Leland, MS, USA). Assessment of selected microalgae extracts with both weeds was carried out in vitro. Prior to in vitro germination, seeds were surface disinfected with 70% ethanol for 7 min, followed by 20% chloride for 7 min, and rinsed twice with sterile distilled water for 10 min. Seeds were then stratified at 4 °C overnight. Seeds were germinated in 1% strength Murashige and Skoog (MS) [30] growth medium agar plates (1.5% *w*/*v*, Bacto agar) under a 16:8 light/dark photoperiod with a light intensity of 50 µmol m^−2^ s^−1^ and a constant temperature of 21 ± 2 °C in a Conviron growth chamber model A1000 (Controlled Environments Inc., Pembina, ND, USA). *Arabidopsis* seed was increased under greenhouse conditions following standard practices. *A. palmeri* seeds were not increased in our laboratory. 

### 2.4. Solvent Extraction of Lyophilized Microalgae Biomass

Prior to biomass harvesting, 30-day-old microalgae batch cultures were spotted onto Lysogeny Broth (LB) agar plates (1.5% *w*/*v*, Bacto agar) and incubated at 37 °C in a New Brunswick™ Innova incubator (Eppendorf, Hauppauge, NY, USA). After 24 h, agar plates were visually inspected for microbial contamination. Only cultures that exhibited no signs of contamination were considered for further studies. Microalgae cultures were harvested by centrifugation (4000 rpm, 15 min). The biomass was flash-frozen in liquid nitrogen and freeze-dried using a FreeZone 2.5 L, −84 °C Benchtop Freeze Dryer (Labconco, Kansas City, MO, USA) under 0.15 mbar vacuum pressure and a collector temperature of −80 °C for 48 h. From the resulting dried biomass, aliquots of 250 mg were transferred to glass vials and extracted separately with 8 mL of four different solvents (hexane, methanol, chloroform, and ethanol). The biomass with each solvent was sonicated for 30 min to favor tissue disruption and then filtered with Whatman No. 2 filter paper. The filtered extracts were dried out in the fume hood for 30 h. When completely dried, the samples were resuspended in 250 µL of the corresponding extraction solvent and used in the different assays. 

### 2.5. Assessment of the Algicidal Effect of Microalgae Extracts

The agar diffusion test was established using strain 3016 as the target organism and TAP agar plates to test the algicidal effect of microalgae extracts. Briefly, different volumes (5, 10, and 15 µL) of the microalgae extracts were added to filter paper disks (Whatman No. 2) of 8 mm diameter, which were allowed to dry out in a fume hood. The paper discs containing the microalgae samples were placed immediately onto the agar plates (50 × 15 mm D, VWR International LLC, Radnor, PA, USA) freshly inoculated with the target microalgae. The strain 3016 (200 µL of OD_750_ 0.5 culture) was spread onto agar plates with sterile borosilicate beads. For the controls, 10 µL of each solvent and 5 µL of kanamycin (100 μg/mL) were applied instead of the algal extracts following the abovementioned procedures. Culture viability controls were set up. Agar plates were incubated, as mentioned above, for seven days. Independent experiments were repeated three times with three technical replicates each.

### 2.6. Assessment of the Nematocidal Effect of Microalgae Extracts

To test the capacity of algal extracts to inhibit *C. elegans* growth, 10–15 L1 larvae nematodes were inoculated in an S-basal medium [29] per replicate. *C. elegans* worms were treated with three volumes of each algal extract (2, 5, and 10 μL) for each solvent extraction. For the negative control, worms were treated with 2, 5, and 10 μL of the corresponding solvent. To analyze the survival rate of L1 larvae, dead and alive worms were visually inspected 24 and 48 h after treatment and counted after 72 h of treatment using a stereomicroscope (Olympus SZX10, Center Valley, PA, USA). Independent experiments were repeated two times with five technical replicates.

### 2.7. Assessment of the Herbicidal Effect of Microalgae Extracts

The bioassay using the different algal extracts was performed in 24-well agar plates, following the above standard procedures. Three different volumes (2, 5, and 10 μL) of the microalgae extract were used for the assay. The corresponding volume was placed onto the agar medium, allowed to dry out, and then *A. thaliana* seeds were sowed. For the negative control, 2, 5, or 10 μL of the corresponding solvent was used instead, and then *A. thaliana* seeds were sowed. Positive controls were implemented using phosphinothricin (15 μg/mL) and kanamycin (50 µg/mL). Seed germination was verified in all experiments by sowing seed onto TAP agar medium. All plates were incubated, as mentioned above. Independent experiments were repeated two times with four technical replicates.

### 2.8. Metabolite Profiling of Microalgae Extracts

Mass spectrometry for untargeted metabolomics of biomass extracts of microalgae strains was performed in a Vanquish Flex Ultra High-Performance Liquid Chromatography (UHPLC) instrument with an Orbitrap Exploris 240 mass spectrometer (MS) (Thermo Fisher Scientific, Waltham, MA, USA). An XBride PREMIER Peptide BEH C18 Waters column (2.1 × 150 mm, 2.5 mm particle size) was used, and the system operated in positive ionization mode with an ESI voltage of 3500V. Biomass extracts were prepared and filtered using OnPu syringe filters PTFE 0.22 μm (Gorzizen, China). The analysis was performed using the AcquireX workflow for the automated generation of the background exclusion list and management of data-dependent acquisition (DDA) and dynamic exclusion (DE). The exclusion list was automatically generated from the matrix blank (methanol) and exported to the data acquisition methods for subsequent analyses. The automatic gain control (AGC) target value was set at 2 × 10^5^ for the entire MS and 5 × 10^4^ for the MS/MS spectral acquisition. The mass resolution was set to 120,000 for full scan MS and 60,000 for MS/MS events and a scan range (*m*/*z*) of 70–800. High-energy collision dissociation (HCD) was performed with a stepped collision energy of 30, 50, and 80%. The inclusion list was generated using a pool of each sample (20 mL of each sample corresponding to each strain) which also served as quality control. The solvents used were HPLC-grade water (solvent A) (W5-4, Thermo Fisher Scientific, Waltham, MA, USA) and HPLC-grade methanol (solvent B) (A452-4, Thermo Fisher Scientific, Waltham, MA, USA), both containing 0.1% formic acid (Sigma-Aldrich, St. Louis, MO, USA). The total LC run time was 25 min and consisted of a gradient with a 0.2 mL/min flow rate. The gradient started in the 5% mobile phase B for 4 min. It was gradually increased to 100% after 13 min. Later, it was kept constant at 100% phase B for 4 min. Phase B was reduced to 5% at min 21 and was maintained in the same condition until min 25 to re-equilibrate the column.

### 2.9. Data Analysis

The acquired data were analyzed using Compound Discoverer v3.3 (Thermo Fisher Scientific, Waltham, MA, USA), a commercial software package developed by Thermo Fisher Scientific to analyze data from their mass spectrometers. LC-MS RAW files were uploaded, and study factors in the form of categorical factors were defined. The untargeted workflow was used, adding the following databases integrated into the CD software: BioCyc, Carotenoids, LipidMAPS, Chemical Biology Department, Max Planck Institute of Molecular Physiology, Marine Drugs, Nature Chemical Biology, NIST, and Pesticide Common Names. This workflow performed retention time alignment, identified compounds using mzCloud (HighChem LLC, Bratislava, Slovakia) and ChemSpider (The Royal Society of Chemistry, Piccadilly, London, UK) databases, and performed ANOVA and Tuckey comparisons statistical analyses. The compound table was filtered by peak quality (peak rating > 5).

To perform a functional analysis of each metabolite and an over-representation analysis of the metabolites present in each sample, we upload their numeric mass (*m*/*z*), retention times (RT) and adj. *p*-values to MetaboAnalyst server (http://www.metaboanalyst.ca/, accessed on 27 June 2023) [31,32] using a mass tolerance of 5.0 ppm. Masses were searched in positive mode, and the enrichment analysis was performed using the Mummichog algorithm version 2.0 (http://mummichog.org/, accessed on 27 June 2023) [33] with a *p*-value cutoff of 1.0 × 10^−5^ and selecting the *A. thaliana* library integrated into MetaboAnalyst. For the enrichment analysis, we selected the chemical structure main-class library [34]. Subsequently, for metabolic pathways analysis we used the *C. vulgaris* (green algae) pathway library from the *Kyoto Encyclopedia of Genes and Genomes* (KEGG) integrated into MetaboAnalyst. An UpSet plot was used to visualize the intersections between metabolites in the different strains using the R package UpSetR [35,36]. The identified metabolites among the different strains and the abundance of intermediates of the isoquinoline alkaloid biosynthesis pathway were compared via heatmap analysis using the R package ComplexHeatmap [37]. In the latter case, the analysis employed the group areas derived from the output table of CD, specifically focusing on the group areas associated with the identified compounds involved in the isoquinoline alkaloid biosynthesis pathway. Principal Component Analysis (PCA) of metabolite profiles was conducted using as input the normalized area values for each technical replicate and each compound with an assigned name from the Compound Discoverer output tables. PCA was conducted in R with the prcomp function from the stats library [36], and the corresponding coordinates for each PCA dimension were plotted using ggplot2 [35]. 

## 3. Results

### 3.1. Aged Cultures of Green Microalgae Produce Metabolites with Algicidal Activity

**Figure 1:**
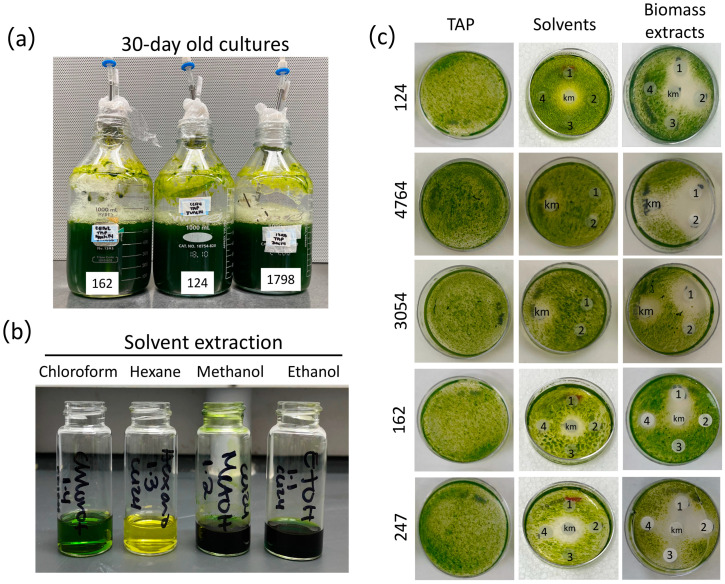
Microalgae biomass extracts preparation and assessment of their algicidal activity. (**a**) 30-day-old representative cultures of *Chlorella* (162 and 1798) and *Chlamydomonas* (124) strains. (**b**) Representative biomass solvent extraction of strain 124 with the four solvents chloroform, hexane, methanol, and ethanol (see Section 2). (**c**) Disk-diffusion assays were conducted to evaluate the impact of biomass solvent extracts [methanol (1), ethanol (2), hexane (3), and chloroform (4)] produced from stationary-phase cultures of *Chlorella* sp. (162), *C. saccharophilum* (247), and *Chlamydomonas* (124, 4764, 3054) strains (see Section 2). The strain *C. sorokiniana* 3016 was used as the target organism. Kanamycin (km, 50 μg/mL) was used as a positive control; TAP Petri dishes show strain growth with no solvents or microalgae extracts.

To investigate the presence of allelochemicals with a potential pesticide effect in microalgae cultures, ten green microalgal strains, four of the genus *Chlorella*, one of the genus *Chloroidium*, and five of the genus *Chlamydomonas*, were evaluated. Upon 30 days of culture under controlled conditions (see Section 2), all strains were tested for contaminants and cell viability, and then the biomass was harvested by centrifugation (Figure 1). Stationary cultures exhibited a color change to dark green pigmentation and no noticeable pH changes (Figure 1a). Although freshly prepared medium was not resupplied to the culture throughout the experiment, cells of all strains were viable after 30 days of culture (Figure S1). Only those cultures free of contaminants were harvested; the biomass was freeze-dried, and metabolites were extracted from the biomass using methanol, ethanol, hexane, and chloroform (Figure 1b and Figure S1). 

**Figure 2:**
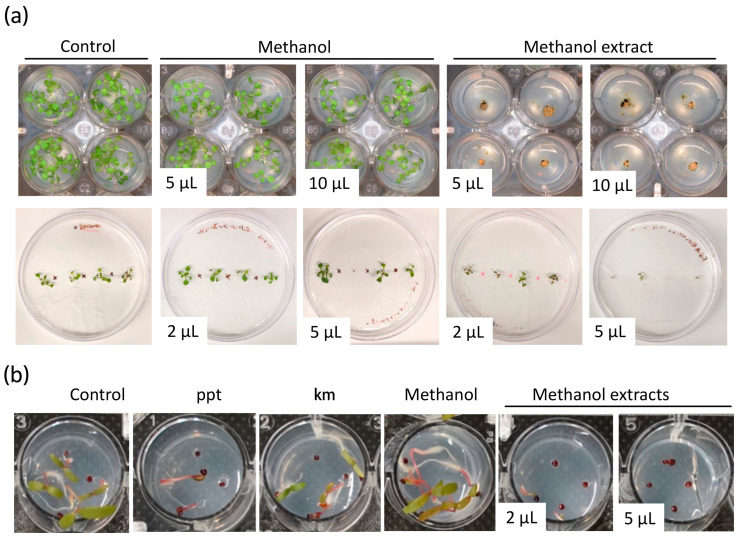
Biomass extracts of *Chlamydomonas reinhardtii* CC124 inhibit the growth of *Arabidopsis thaliana* and *Amaranthus palmeri*. (**a**) *A. thaliana* and (**b**) *A. palmeri* growth-inhibition assays using methanol biomass extract of strain 124. In (**a**), the top panel shows a view of the aerial part of *Arabidopsis* seedlings grown in 24-well plates. The bottom panel shows *Arabidopsis* seedlings grown vertically onto the agar plates. Control treatments indicate seed germination on MS growth medium without solvent or biomass microalga extract. Phosphinothricin (ppt, 15 μg/L) and kanamycin (km, 50 μg/mL) were used as the positive controls. The volume in the pictures indicates the volume of biomass extract utilized for the assay.

We first tested the activity of the biomass extracts in inhibiting the growth of a fast-growing photosynthetic organism, i.e., other microalgae. To this end, we established a paper disk-diffusion assay using different volumes of biomass extracts to determine their capacity to inhibit the growth of strain 3016 as the target organism. This microalga strain was selected based on its remarkable growth rate, with a duplication time of 2.88^−1^ under similar growth conditions to those employed in our study (temperature and nutrient concentration) [38]. The four extracts prepared from each strain were tested on the same plate along with kanamycin as a positive control, whereas solvents alone were tested on a separate plate. As expected, a sharp halo of inhibition was observed for the kanamycin (13 mm diameter), whereas no negative effects were observed for the treatment with solvents only (Figure 1c and Figure S2). We found that the several microalgae biomass extracts generated using the four solvents compromised the growth of strain 3016 with different efficacies (Figure 1 and Figure S3, Table S1). In some cases, we observed that only methanol and ethanol extracts from the same strain effectively inhibited microalgal growth, as observed in the halo of inhibition. In contrast, in other cases, chloroform extracts also presented an algicidal effect (Figure 2 and Figure S3, Table S1). For instance, methanol and ethanol extracts of strains 124, 4764, and 247 presented algicidal activity. In the case of strain 1798, chloroform and methanol extracts presented algicidal activity, whereas only methanol extracts presented the same activity in strains 162 and 3054 (Figure 1 and Figure S3, and Table S1). These data suggest that metabolites with different properties may underlie the growth-inhibiting activity over strain 3016.

### 3.2. Crude Extracts of Green Microalgae Control Seed Germination of an Aggressive Glyphosate-Resistant Weed

*Arabidopsis* is a small weedy plant that has been adopted as a model for many studies in plant biology. Thus, it is a good model to test the herbicidal activity of microalgal extracts. We then established a simple and effective method of germinating *Arabidopsis* seedlings in 24-well plates as an initial step to assess the potential herbicidal activity of the microalgae extracts. Different volumes (2, 5, and 10 μL) of each extract were applied to the agar medium, and *Arabidopsis* seeds were sowed. Wells with solvent and MS growth medium only were used as controls and evaluated after seven days (see Section 2).

We identified extracts from the different microalgal strains which affected seedling growth or completely aborted seed germination (Figure 2 and Figure S4, and Table S1). The inhibitory activity of the microalgae biomass extracts was variable based on the solvent used for extraction and the volume of crude extract utilized for the assay (Table S1). Remarkably, the biomass extract of some strains showed a noticeable effect on seedling growth even with the lowest volume used, 2 μL (Figure 2). For instance, 5 μL of methanol extract from strain 124 affected seedling growth, whereas 5 μL of ethanol extract from the same strain aborted or inhibited seed germination (Figure 2 and Figure S5). Interestingly, in the case of strains 1602 and 247, extracts prepared with the four different solvents showed an inhibitory effect of *Arabidopsis* seed germination (Figure S4 and Table S1). Seeds sowed on MS and MS + solvent controls germinated and grew normally. Similar experiments with methanol extracts of strain 124 growing *Arabidopsis* seedlings vertically onto the agar showed that 5 μL were effective at impeding shoot and root growth (Figure 2a and Figure S5). Suggesting that the type of metabolites and their concentration in this biomass extract are effective enough to control weeds.

Glyphosate-resistant *A. palmeri* biotypes are among the most aggressive weeds that infest cotton, peanut, and soybean fields in the U.S. and other regions of the world [39]. They show an aggressive growth habit and prolific seed production. Glyphosate application rates exceeding the average use rate of 0.84 kg ae ha^−1^ by three and twelve times allow control of glyphosate-resistant *A. palmeri* biotypes in the field by only 17 and 82%, respectively [40]. To evaluate the efficacy of microalgae extracts in controlling the growth of glyphosate-resistant *A. palmeri*, methanol biomass extract from strain 124 that was effective at controlling *Arabidopsis* shoot and root growth at low volumes was selected for in vitro testing. Phosphinothricin and kanamycin were used as positive controls. These two compounds are known to affect plant growth and are widely used as selectable markers. Phosphinothricin was more effective than kanamycin in controlling the growth of glyphosate-resistant *A. palmeri* (Figure 2b). Importantly, we found that 2 μL of the methanol extract compromised seed germination and seedling growth and was as effective as the phosphinothricin concentration used in this study. Still, about 10% of the tested seeds were able to germinate under both treatments. Complete seed germination abortion was observed with 5 μL of the methanol extract, which was more effective than phosphinothricin and kanamycin in controlling the growth of glyphosate-resistant *A. palmeri* (Figure 2b). The carrier solvent methanol (solvent control) did not negatively affect seed germination and seedling development. Well plates were maintained for over 20 days under the same conditions to investigate whether the seed could still germinate, possibly due to the degradation of the bioactive molecules in the microalgae extracts or the lack of effectiveness of the volume used. We observed that about 20% of the seeds treated with 2 μL of methanol extract of strain 124 were able to germinate and grow (Figure S6). However, in the case of the treatment with 5 μL of biomass extract, only a few seeds showed radicle emergence, but none of them thrived even after 20 days of treatment (Figure S6). These findings highlight the potential of the biomass extract of strain 124 as an effective herbicide against *A. palmeri* and further suggest its potential as a natural alternative to conventional herbicidal agents. 

### 3.3. Crude Extracts of Green Microalgae Kill C. elegans L1 Larvae Nematode

**Figure 3:**
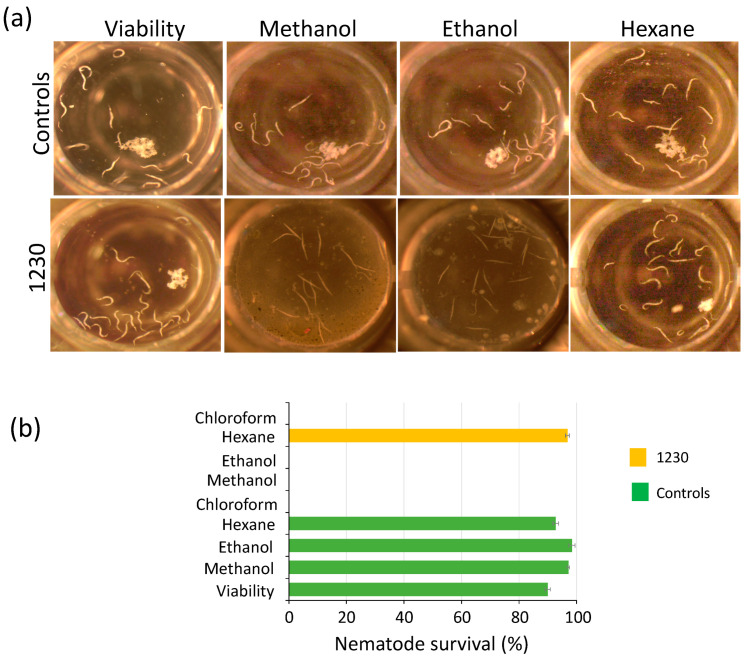
Biomass extracts of *Chlorella sorokiniana* UTEX 1230 kill *Caenorhabditis elegans* L1 larvae. (**a**) The biomass extracts of strain 1230 were tested for killing *C. elegans* L1 larvae. Living nematodes are visible as curved white strings, whereas dead nematodes appear as straight strings after 72 h of treatment. (**b**) Nematode survival (%) of experiments in (**a**). Survival percentage is shown as the mean and standard error of all nematodes in the well (*n* = 4) (see Section 2).

Weeds represent a reservoir of pests and diseases in the field, further complicating pest management. Pathogenic nematodes belonging to diverse genera and species represent a serious threat to a wide range of hosts. Some of the most damaging nematodes are root-knot (*Meloidogyne* spp.), cyst (*Heterodera* and *Globodera* spp.), and reniform (*Rotylenchulus reniformis*) [41]. In this context, plant–host resistance has resulted in an effective and eco-friendly control strategy for pathogenic nematodes. However, nematodes often form disease complexes with fungi like *Fusarium*, thus further complicating pest management. Therefore, the use of several pesticides is often an unavoidable practice. We assessed the potential of the microalgal extracts to disrupt the growth of the free-living nematode *C. elegans* because it provides a suitable model to investigate their potential to control or kill pathogenic nematodes that infest crop fields. We used 96-well plates for these assays, placing 12–16 L1 larvae per well for testing the extracts (see Section 2). The potential toxic effect of the solvents used as metabolite carriers (methanol, ethanol, hexane, and chloroform) was tested. Among these solvents, only chloroform exhibited toxic effects on the nematode, while the other solvents (methanol, ethanol, and hexane) showed no negative effects (Figure 3). We found that biomass extracts killed L1 nematodes 72 h after treatment. Dead nematodes were straight, not motile, and did not produce offspring after the treatment (Figure 3 and Figure S7, Tables S1 and S2). Methanol and ethanol biomass extracts of *Chlorella* strains 162, 1230, 1798, 1602, and *Chloroidium* species 247 showed lethal effects on the nematode, as 100% died after 72 h, while hexane extracts of all microalgae strains had no toxic effects against *C. elegans* (Figure 3 and Figure S7). Interestingly, when exposed to ethanol biomass extract of strain 4764, 100% of the larvae died, whereas 57% survived under methanol treatment (Figure S7, Table S2). An inverse scenario was observed for methanol and ethanol extracts of strain 125 that caused the death of 100% and 38% of the larvae, respectively (Figure S7, Table S2). In contrast, methanol and ethanol extracts of strains 124 and 3054 effectively killed 100% of *C. elegans* larvae (Figure S7, Table S2). These results suggest the potential presence of molecules that act as nematicides that can be employed as a natural alternative to synthetic pesticides.

### 3.4. Non-Targeted Metabolomics Analysis of Microalgae Crude Extracts Suggests Highly Diverse Chemical Classes Potentially Involved in Pesticide Activities

A nontargeted metabolomics analysis of methanolic extracts was performed to gain insights into the diversity of chemical classes of metabolites in the microalgae extracts and molecules potentially associated with the algicidal, herbicidal, and nematocidal effects observed in this study. Methanolic extracts of six strains, namely 3054, 124, 162, 1798, 1602, and 1230, were analyzed by UHPLC-MS (see Section 2). The metabolome of each strain consisted of chromatographic peaks with signal-to-noise ratio > 3.0 and peak quality > 5.0 in the normalized chromatographic area. The overall composition of the metabolome was determined through spectral searches against mzCloud MS/MS and additional databases (see Section 2). The metabolomic dataset for strains 3054, 124, 162, 1798, 1602, and 1230 consisted of 3372, 4206, 3405, 3429, 3498, and 3986 compounds, respectively (Dataset S1). These findings highlight the comprehensive nature of our metabolomic profiling, providing insights into the rich chemical composition of the tested extracts and strains.

To obtain an overview of the similarities or differences between the pool of metabolites for the different microalgae extracts, we did a Principal Component Analysis (PCA) for each sample and each injection replicate. A plot of dimensions 1 and 2 (PC1 and PC2, Figure S8a) showed that samples from strains 1230 and 124 have very distinct metabolite profiles, while 1798, 1602, 162, and 3054 cluster together, suggesting they have similar profiles, with 1798 and 1602, and 162 and 3054 being almost indistinguishable from each other. However, when dimensions 3 and 4 were plotted (Figure S8b), none of the samples from different strains clustered together, suggesting different metabolite profiles. Differences among metabolite profiles became evident when we plotted dimensions 1 and 2 of a PCA analysis of the 100 most abundant metabolites in each microalga extract (Figure S8c). This indicates that the metabolites present in strains 1230 and 124 are distinct and that those of 1798, 1602, 162, and 3054 have similarities in general terms but that important differences exist between the identity of the most abundant metabolites in each extract. 

**Figure 4:**
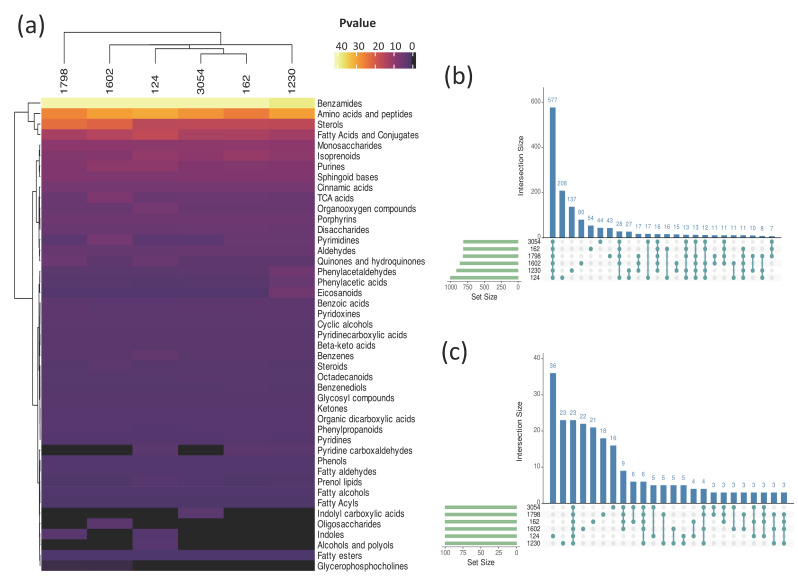
Enriched chemical categories of metabolites in methanol biomass extracts of aged cultures of six microalgae strains. (**a**) Heatmap representing enriched chemical categories of metabolites present in methanol extracts of biomass from strains 1798, 124, 162, 3054, 1230, and 1602 after 30 days of culture, based on MetaboAnalyst analysis (see Section 2). The statistical significance key, −log(Pvalue), indicates the threshold for enrichment. (**b**) Upset analysis of all metabolites in (**a**). (**c**) Upset analysis of the 100 more abundant metabolites in each microalga extract from (**a**), based on normalized relative abundance. Set Size indicates the number of named compounds for each species. Intersection size indicates the number of compounds present in the corresponding intersection.

To further investigate the chemical classes to which the metabolites profiled belong, all databases were analyzed using the MetaboAnalyst server (http://www.metaboanalyst.ca/, accessed on 27 June 2023) [31,32] and the Mummichog algorithm (http://mummichog.org/, v2, accessed on 27 June 2023) [33]. Because this analysis utilized a library of known molecules from *A. thaliana*, the pool of annotated metabolites for strains 3054, 124, 162, 1798, 1602, and 1230 was reduced to 26.8%, 27.4%, 33.1%, 26.1%, 26.4%, and 27.6%, respectively, of the total metabolites detected. This suggests that all those molecules not assigned a chemical class have not been detected in higher plants yet and may be unique to microalgae. We found several enriched metabolite categories in all the methanolic extracts analyzed, including benzamides, amino acids and peptides, fatty acids and conjugates, sterols, and isoprenoids (Figure 4a, Tables S3–S8). Interestingly, some metabolite categories were exclusive to certain strains. For instance, the indolyl carboxylic acids category was exclusive to 3054; the alcohols and polyols category was exclusive to strain 124, and the oligosaccharides category was exclusive to strain 1602; the indoles category was enriched in 124 and 1798, and pyridine carboxyaldehydes were enriched in 162, 124, and 1230 (Figure 4a). An UpSet analysis was performed to determine which metabolites are common and unique to the six strains. A total of 577 metabolites were shared among all the strains, and 208, 137, 80, 54, and 44 metabolites were unique to strains 124, 1230, 1602, 1798, 162, and 3054, respectively (Figure 4b, Dataset S1). An UpSet analysis of the 100 more abundant metabolites in each methanol extract revealed that only 23 metabolites are shared between all strains, whereas 36, 23, 22, 21, 18, and 16 are exclusive to strains 124, 1230, 1602, 162, 1798, and 3054, respectively (Figure 4c, Dataset S1). The more abundant shared metabolites included (9Z)-9-Octadecenamide, Traumatin, Umbelliferone, Xestoaminol, 4-Linoleamide, and palmitamide. 

**Figure 5:**
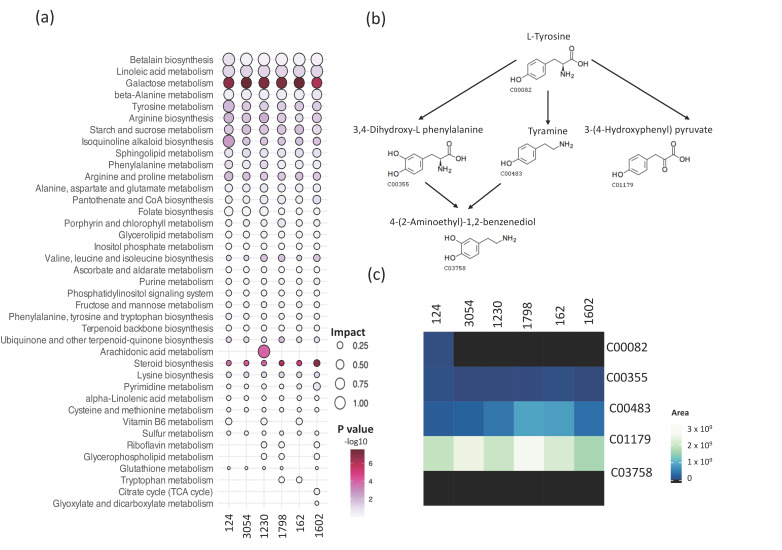
Analysis of metabolic pathways represented in the metabolic profiles of methanol biomass extracts of aged cultures of six microalgae strains. (**a**) Metabolic pathway enrichment and topology analyses using MetaboAnalyst (v5.0) and KEGG (see Section 2). Size is proportional to the impact value, and the key, −log10 (*p* value), indicates the threshold for statistically significant enrichment of a metabolic pathway. Only pathways with an impact value of 0.1 or higher in at least one species are shown. (**b**) Schematic representation of a portion of the isoquinoline alkaloid biosynthesis pathway based on the KEGG database and KEGG reference number for the intermediates. (**c**) Heatmap of the normalized peak areas of intermediaries in (**b**), detected in the metabolomic profiling of the methanol extracts from biomass of strains 1798, 124, 162, 3054, 1230, and 1602.

The *Kyoto Encyclopedia of Genes and Genomes* (KEGG) and MetaboAnalyst were then used to integrate metabolic pathway enrichment and topology analyses. Metabolic pathways with impact values greater than 0.1 were analyzed, obtaining 39 pathways (Figure 5, Dataset S1). Pathways enriched in all six analyzed strains were thirty-one out of thirty-nine, whereas two metabolic pathways were enriched exclusively in strain 1602 and one in strain 1230 (Figure 5a). Betalain biosynthesis followed by linoleic acid were the pathways with the higher impact value in all strains. Additionally, the pathways with higher impacts (>0.5) in at least one strain are beta-Alanine, galactose, tyrosine, isoquinoline alkaloid biosynthesis, and arachidonic acid metabolism. Importantly, molecules related to some of these pathways protect plants against several stresses or possess bioactivity. For instance, betalains are secondary metabolites reported to protect plants against abiotic stresses such as low and high temperature, metal ions, light, and exposure to polyamines, spermidine, and putrescine [42,43] and play a crucial role in defense against pathogenic fungi [44]. Another interesting pathway represented in the metabolite profiles of the six strains is isoquinoline alkaloid biosynthesis. Isoquinoline alkaloids are a class of *N*-based heterocyclic compounds synthesized from L-tyrosine that have been described to possess a broad range of bioactivities such as antibacterial, antifungal, antiviral, antiparasitic, and insecticidal [45]. The isoquinoline alkaloids synthesis pathway is complex, and importantly, several of the intermediates were found in our metabolite profiles, including 3,4-Dihydroxy-L-phenylanaline, Tyramine, and 3-(4-hydroxyphenyl) pyruvate (Figure 5b). The LC-MS and CD analysis yielded valuable insights into the relative abundance of intermediates in the synthesis pathway of isoquinoline alkaloids. The normalized peak areas revealed that 3-(4-Hydroxyphenyl) pyruvate was the most abundant intermediate, followed by Tyramine, and then 3,4-Dihydroxy-L-phenylalanine. However, L-tyrosine was detected only in samples of strain 124 (Figure 5c). These data provide valuable insights into the chemical classes of metabolites in the biomass extracts and the related metabolic pathways that may contribute to the pesticide activity of *Chlorella*, *Chlamydomonas*, and *Chloroidium* strains. Furthermore, they represent valuable resources to explore the use of these strains for biocontrol strategies and molecule discovery.

## 4. Discussion

Microalgae are recognized as prolific producers of secondary metabolites encompassing a diverse range of compounds such as aldehydes, alkaloids, fatty acids, peptides, and carotenoids [46]. These metabolites are often accumulated within the microalgae cells or secreted into the surrounding environment during growth [11,15,16,47,48]. Some of these secondary metabolites have been described to possess growth-inhibiting properties. Previous screening studies primarily focused on the study of Cyanobacteria, particularly species belonging to *Anabaena, Calothrix*, *Fischerella*, *Nostoc*, *Oscillatoria*, and *Syctonema*, in the search for algicidal metabolites [49]. However, investigations on green microalgae species and their potential as sources of biopesticides remain largely untapped. In this study, we tested the biomass of green microalgae of the *Chlamydomonas*, *Chlorella*, and *Chloroidium* genera for bioactive compounds with algicidal, herbicidal, and nematocidal activities. Our results revealed that the tested microalgae extracts exhibited inhibitory effects against the three target organisms employed in this study, i.e., *C. sorokiniana*, *A. thaliana*, and *C. elegans* (Figure 1, Figure 2 and Figure 3, Tables S1 and S2). These results suggest that the microalgae cells produce compounds toxic to these organisms, possibly allelochemicals, in response to the prolonged period of culture. Similar studies have identified algicidal activity in aged cultures of different *C. reinhardtii* and *C. vulgaris* strains, and the *Volvocaceae* family beyond 20 days after inoculation [24,26]. The authors attributed this growth-inhibiting activity to a combination of fatty acids that might act as allelochemicals produced in response to the state known as conditional senescence resulting from nutrient limitation [50,51]. Humby et al. (2013) [51] demonstrated that when *C. reinhardtii* is grown in TAP medium under mixotrophic conditions, similar conditions employed in our study, nutrient depletion occurs as early as seven days after inoculation. Phosphorus and nitrogen, both essential nutrients, were the first nutrients to be exhausted [51]. Although we did not determine nutrient levels in our harvested cultures, conditional senescence might cause stress-inducing secondary metabolite production in the 10 strains we employed.

Early studies with *C. vulgaris* reported that this microalga’s aged cultures (2–3 weeks) possess algicidal activity. Although the responsible compound was not isolated and further characterized, it was named Chlorellin, and described as a mixture of fatty acids and hydrocarbons [24,52,53]. Similarly, 20-day-old cultures of *C. reinhardtii* excrete a fatty acid mixture that inhibits the growth of *Haematococcus lacustris* (formerly *Haematococcus pluvialis*) [25]. Untargeted metabolomics analysis using UHPLC-MS enabled metabolite profiling of the methanolic extracts of six of ten microalgae strains screened in our study. We identified a wide diversity of metabolites produced by each species. The metabolites detected belong to 45 different chemical classes, suggesting a highly diverse chemical profile. Among the more abundant metabolites across all strains, notable compounds include (9Z)-9-Octadecenamide, Traumatin, Umbelliferone, Xestoaminol, 4-Linoleamide, and palmitamide. Umbelliferone, also known as 7-hydroxycoumarin, belongs to the coumarin family and has been shown to possess antimicrobial properties, functioning particularly as an antibacterial [54] and antifungal agent [55], as well as exhibiting antioxidant properties [56]. However, it has not been tested as an inhibitor of growth or seed germination. The fact that another member of the coumarin family, imperatorin, isolated from the plant *Esenbeckia yaxhoob*, inhibits radicle growth in *L. esculentum* and seed germination in *L. sativa* and *L. esculentum* [57] suggests that umbelliferone, present in the methanol extract of strain 124 effective to control *A. palmeri* seed germination, exerts an herbicidal effect as well. Interestingly, Razavi (2009) [58] reported the cytotoxic effects of the methanolic extract derived from *Z. absinthifolia* fruits containing coumarins on *L. sativa*. This extract was found to significantly reduce seed germination, as well as impede shoot and root growth in lettuce [58]. These suggest that green microalgae are significant sources of coumarin molecules with important biotechnological applications.

The extract of the strain 162 contained three known bioactive compounds Z)-9-octadecenamide, palmitamide, and cinnamic acid. (9Z)-9-octadecenamide, the amide derivative of oleic acid obtained from the marine sponge *Dendrilla nigra*, and palmitamide, another fatty acid amide, have been reported to have antimicrobial properties [59]. Cinnamic acid, a natural aromatic carboxylic acid widely distributed in plants and microalgae [60,61], was present in higher amounts in extracts of strain 162 than in other strains. Numerous studies have reported various beneficial effects of this carboxylic acid, including antioxidant, antimicrobial, and anti-inflammatory properties [60]. Moreover, cinnamic acid has been shown to induce oxidative stress and cause root cell death in cucumber seedlings, as it enhances the production of reactive oxygen species (ROS) and promotes lipid peroxidation, destroying cell membranes [62]. This suggests that the pesticide activities we observed in a single extract might be due to the concerted action of multiple metabolites, acting as a cocktail of molecules against diverse organisms. 

Metabolite profiling was combined with enriched metabolic pathways identification to gain insights into their functional significance. We identified 31 pathways with an enrichment ratio greater than 0.1 common to the six analyzed strains (Figure 5a), including betalain biosynthesis, linoleic acid metabolism, galactose metabolism, tyrosine metabolism, isoquinoline alkaloid biosynthesis, porphyrin and chlorophyll metabolism, and sphingolipid metabolism. However, strain-specific enrichment was observed, such as the citrate cycle (TCA cycle) and glyoxylate and dicarboxylate metabolism pathways in strain 1602, and the arachidonic acid metabolism pathway enriched in strain 1230. Additionally, specific pathways, including vitamin B6 metabolism, riboflavin metabolism, glycerophospholipid metabolism, and tryptophan metabolism, were found to be present only in two or three strains, indicating that the associated metabolites may confer distinct properties to these specific strains. The linoleic acid metabolism and isoquinoline alkaloid biosynthesis pathways are of particular interest, which were common to all analyzed strains. Prior studies have reported that a mixture of fatty acids, including linoleic and alpha-linolenic acids, can inhibit the growth of other microalgae or induce autoinhibition [11,24,25]. Additionally, herbicidal activity against *Lemna minor* (*phylum Magnoliophyta*) has been attributed to 2,5-dimethyldodecanoic acid, a fatty acid produced by the cyanobacterium *Lyngbya aesturii* (*Cyanobacteria*) [63]. As part of the linoleic acid metabolism pathway, we detected fatty acids such as linoleic acid, alpha-linolenic acid, and gamma-linolenic acid in the metabolic profiles of all strains (Dataset S1), which suggests these fatty acids may contribute to the pesticidal activity detected. It has been reported that many microalgae species important to biofuel production due to their high fatty acid and neutral lipid content also release some fatty acids that may reach high enough levels to cause self-inhibition or toxicity to the strains of interest or other strains. Therefore, ultimately hurting biomass productivity. Such negative effects of fatty acids are attributed to the damage to the plasma membrane, which may cause leakage of cellular content and cellular lysis [64].

Another category of interesting metabolites is the isoquinoline alkaloids biosynthetic pathway, which was detected in all six analyzed strains (Figure 5). Isoquinoline alkaloids have been reported to possess diverse activities such as antiviral, antifungal, and enzyme inhibitory activities [45,65]. In a study with *Chelidonium majus*, methanol extracts exhibited antimicrobial activity; the extract contained seven different isoquinoline alkaloids that were toxic to the Gram-positive pathogen *S. aureus* [66]. Additionally, the antifungal activity of twelve isoquinoline alkaloids was evaluated against eight plant pathogenic fungi, demonstrating different strengths of fungicide activities. However, to our knowledge, the effect of isoquinoline alkaloids against microalgae, nematodes, or plants is unknown. In our study, we detected intermediates of the isoquinoline alkaloids biosynthesis (Figure 5), such as L-tyrosine (C00082), 3,4-Dihydroxyl-L-phenylalanine (C00355), tyramine (C00483), and 3-(4-Hydroxyphenyl) pyruvate (C01179), the latter being more abundant in all strains (Figure 5). Three of these metabolites (C00355, C00483, and C01170, Figure 5) were identified in the remaining strains. Importantly, methanol extracts of 124, 3054, 125, 4764, 1798, 1230, and 1602 strains effectively killed *C. elegans* in our study. The fact that the enrichment pathway analysis revealed that the analyzed strains (124, 3054, 1230, 162, 1602, and 1798) produce metabolites related to the isoquinoline alkaloid biosynthesis pathway suggests that molecules related to this pathway could be responsible for the nematocidal effect. This is further supported by a filed patent that protects the use of isoquinoline alkaloids as part of a formulation with insecticidal, acaricidal, and nematocidal action [67]. Interestingly, a previous study demonstrated the nematocidal effect of 15-day-old cultures of the cyanobacterium *Nostoc oryzae* (formerly *Anabaena oryzae*) (*Cyanobacteria*) and the two green algae *C. vulgaris* and *Tetradesmus obliquus* (formerly *Scenedesmus obliquus*) against the nematode *M. incognita* in banana plants. However, the specific compound responsible for this effect remain uncharacterized [68]. 

To the best of our knowledge, there are no reports of using green microalgae as sources of growth-inhibiting substances for weed control. Only a few studies have been conducted to assess specifically cyanobacterial toxins as herbicides [48,69,70]. We demonstrated that methanolic extracts of strain 124 effectively control the germination and growth of a glyphosate-resistant *A. palmeri*, suggesting the presence of molecules with herbicidal activity. Glyphosate, an extensively utilized nonselective and broad-spectrum herbicide, is a phosphonic acid derived from the reaction between the methyl group of methylphosphonic acid and the amino group of glycine (N-[phosphonomethyl]glycine) [71]. This herbicide targets the enzyme 5-enolpyruvylshikimate-3-phosphate (EPSP) synthase [71], thus functioning as an inhibitor of amino acid synthesis. As a result, the levels of aromatic amino acids are depleted, leading to the disruption of biosynthetic metabolic pathways and the eventual demise of the plant [72]. In general, glyphosate resistance has been reported to be acquired by several weeds due to different mechanisms, classified as target-site resistance (single base pair alteration, multiple base pair alteration, and gene amplification) and nontarget site resistance (enhanced metabolism and translocation). When target-site resistance occurs, an alteration of the target enzyme EPSPS which confers glyphosate resistance occurs, or an increased enzyme level is produced by gene amplification that increases tolerance to this herbicide. On the other hand, nontarget site resistance refers to a condition where the target enzyme remains susceptible to glyphosate, but a specific mutation has conferred a mechanism that decreases the amount of the herbicide which reaches the EPSPS enzyme [73]. The fact that the methanolic extract of strain 124 controlled the germination and growth of this weed suggests the presence of molecules showing a different mode of action. Interestingly, we detected widely diverse secondary metabolites in the methanolic extracts analyzed, and only ~26–33% were annotated following the conventional LS-MS libraries or enzymatic pathways, suggesting that microalgae produce novel yet-to-be-characterized metabolites. Therefore, these novel metabolites or pathways can potentially be the source of the inhibitors and should be studied in more detail in the search for biopesticide agents. An interesting alternative to biomass microalgae in search of metabolites with pesticidal activities is to test the supernatants as they contain all compounds released by the microalgae cells into the growing environment. After prolonged periods of culture, these allelopathic compounds tend to accumulate in the growth media, thus providing an additional source of bioactive compounds.

## 5. Conclusions

In conclusion, our study highlights the algicidal, herbicidal, and nematocidal activities of biomass extracts derived from microalgae of the *Chlamydomonas*, *Chlorella*, and *Chloroidium* genera. The observed toxic effects on various biological targets coupled with the characterization of the metabolite profiles, potential metabolites responsible for the pesticide activities, and their associated metabolic pathways provide valuable insights into the potential bioactive compounds identified and their underlying mechanisms. Further investigations into these extracts, their composition, and the metabolic pathways involved hold promise for developing more effective and eco-friendly biopesticides with novel bioactive molecules for agriculture. Moreover, future studies employing targeted metabolomic approaches, isolation, and characterization of the specific metabolites would further enhance our understanding of their bioactivity. 

## Data Availability

Data is contained within the article or Supplementary Material.

## Supplementary Materials

The following supporting information can be downloaded at: https://www.mdpi.com/article/10.3390/biology12081149/s1, Figure S1. (a) Cultures of green microalgae strains on the inoculation day using TAP growth media. (b) Petri dishes showing the bacterial contamination tests of 30-day-old cultures of strains 3054, 1798, 4764, 124, 4533 and 162 using LB medium; (*) indicates contaminated culture. (c) Viability tests of microalgae cells after 30 days of culture using TAP agar plates. (d) Vials with freeze-dried biomass (250 mg) of the cultured strains after 30 days. All pictures are representative of the different experiments and tests conducted. Figure S2. Solvents do not produce cytotoxic effects on *Chlorella sorokiniana* UTEX 3016 growth. Three different volumes, 5, 10, and 15 μL of each solvent [methanol (1), ethanol (4), hexane (3), and chloroform (2)], were tested. The lack of effect of solvents was also assessed. Kanamycin (km, 50 μg/mL) was used as a positive control. Pictures represent technical triplicates of the experiments. The control illustrates the growth of strain 3016 onto TAP agar Petri dishes without solvents. Figure S3. Biomass extracts of *Chlamydomonas*, *Chlorella*, and *Chloroidium* strains inhibit the growth of *C. sorokinana* UTEX 3016. Disk-diffusion assay was conducted to evaluate the impact of biomass solvent extracts [methanol (1), ethanol (2), hexane (3), and chloroform (4)] produced from stationary-phase cultures of *Chlamydomonas* (125 and 4533), *Chlorella* (1602, 1798, and 1230), and *Chloroidium* (247) strains (see Section 2). The strain *C. sorokiniana* UTEX 3016 was used as the target organism. Kanamycin (km, 50 μg/mL) was used as a positive control; TAP agar Petri dishes show strain growth with no solvents or microalgae extracts. Independent experiments were repeated three times with three technical replicates. Figure S4. Biomass extracts of *Chlamydomonas*, *Chlorella*, and *Chloroidium* strains present herbicidal activity. Assessment of biomass extracts of *Chlorella* (162, 1230, 178, 1602), *Chloroidium* (247), and *Chlamydomonas* (3054, 4764, 125, 4533) strains for *Arabidopsis* growth and seed germination inhibition. Control conditions using MS growth medium with no microalgae extracts and solvents only were used to test seed germination. Figure S5. Assessment of biomass extracts of strain *C. reindhardtii* CC124 for *Arabidopsis* growth and seed germination inhibition with two volumes of the extract (2 μL and 5 μL). Control conditions using MS growth medium with no microalgae extracts and solvents only were used to test seed germination. Figure S6. Methanol extract of *C. reinhardtii* CC124 inhibits the growth of *A. palmeri*. Top view of *A. palmeri* 20-day-old seedlings treated with methanol biomass extracts of strain 124. Rows represent technical replicates of representative plants. Control indicates growth medium without solvent or biomass extract. Phosphinotricin (ppt, 15 μg/L) and kanamycin (km, 50 μg/mL) were used as positive controls. Volume indicates the volume of biomass extract utilized. Figure S7. Biomass extracts of *Chlamydomonas*, *Chlorella*, and *Chloroidium* strains present nematocidal activity. Assessment of the toxic effect of biomass extracts from *Chlorella* (162, 1230, 1798, 1602), *Chloroidium* (247), and *Chlamydomonas* (3054, 4764, 125, 4533, 124) strains on *C. elegans*. Living nematodes are visible as curved white strings, and dead nematodes appear as straight strings after 72 h of treatment. Control conditions with solvents only and no microalgae extracts were used to evaluate their effect on nematode viability. Figure S8. Principal Component Analysis of the named compounds (a,b) and 100 more abundant metabolites (c) from our analysis of the metabolite profiles of strains 1230, 1602, 1798, 124, 3054, and 162 with Compound Discoverer. The normalized areas of named compounds were used for the analysis. Each dot represents a technical replicate. Table S1. Pesticide activities of microalgae biomass extracts tested against *C. sorokiniana* UTEX 3016 (*Cs*), *A. thaliana* (*At*), and *C. elegans* (*Ce*). Table S2. *C. elegans* L1 larvae survival (%) after 72 h of treatment with microalgae biomass extracts. Table S3. Major chemical classes of metabolites identified in methanol extract of strain *C. sorokiniana* UTEX 1230. Table S4. Major chemical classes of metabolites identified in methanol extract of strain *C. sorokiniana* UTEX 1602. Table S5. Major chemical classes of metabolites identified in methanol extract of strain *Chlorella* sp. ‘anitrata’ 1798. Table S6. Major chemical classes of metabolites identified in methanol extract of strain *C. reinhardtii* *CC*124. Table S7. Major chemical classes of metabolites identified in methanol extract of strain *C. reinhardtii* *CC*3054. Table S8. Major chemical classes of metabolites identified in methanol extract of strain *Chlorella* sp. UTEX EE162. Dataset S1. Metabolite profiles of methanol extracts of *Chlorella*, *Chloroidium*, and *Chlamydomonas* strains, UpSet analysis, and metabolic pathways.
