# Supplementary material for: Bioactive Compounds with Pesticide Activities Derived from Aged Cultures of Green Microalgae
Source: Biology (Basel). 2023 Aug 19;12(8):1149. doi: 10.3390/biology12081149 (PMC10452921; doi:10.3390/biology12081149)
Supplement: Supplementary file 1 [file biology-12-01149-s001.zip › Supplementary materials.pdf]

Supplementary Information

Bioactive compounds with pesticide activities derived from aged cultures of green microalgae

Alethia A. Brito-Bello[1], Damar Lopez-Arredondo[1,*]

[1] Institute of Genomics for Crop Abiotic Stress Tolerance, Department of Plant and Soil Science, Texas Tech University, Lubbock, TX 79409, USA.

*Correspondence: Damar.Lopez-Arredondo@ttu.edu (D. Lopez-Arredondo)

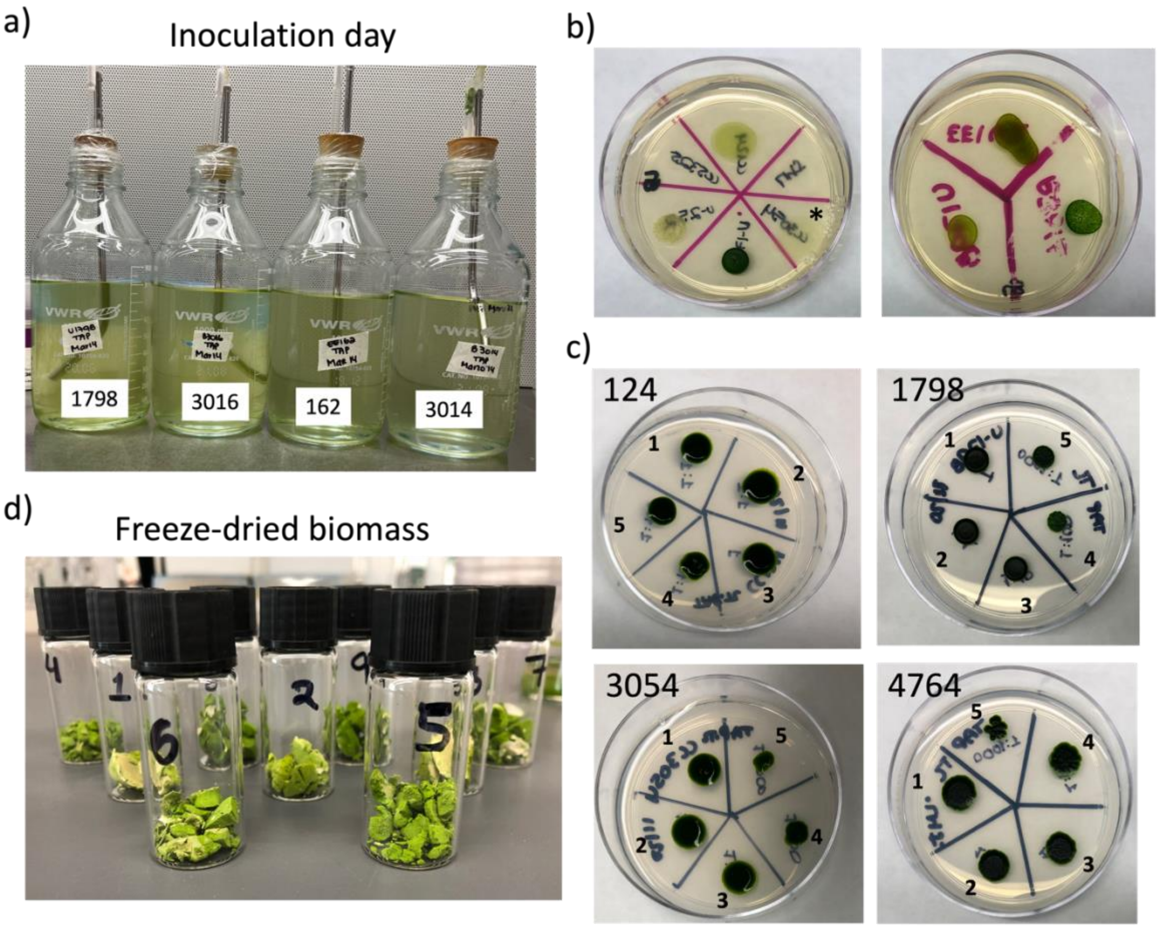

Figure S1. **(a)** Cultures of green microalgae strains on the inoculation day using TAP growth media. **(b)** Petri dishes showing the bacterial contamination tests of 30-day-old cultures of strains 3054, 1798, 4764, 124, 4533, and 162 using LB medium; (*) represents contaminated culture. **(c)** Viability tests of microalgae cells after 30 days of culture using TAP agar plates. **(d)** Vials with freeze-dried biomass (250 mg) of the cultured strains after 30 days. All pictures are representative of the different experiments and tests conducted.

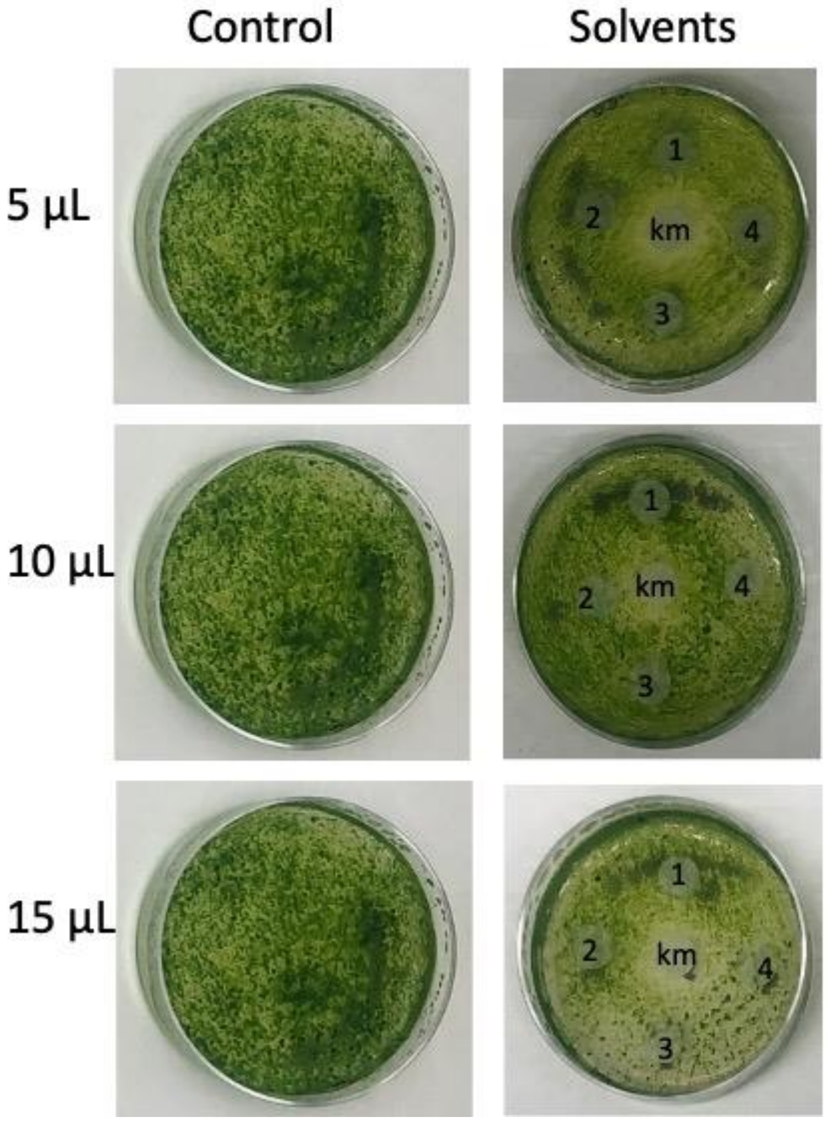

Figure S2. Solvents do not produce cytotoxic effects on *C. sorokiniana* UTEX 3016 growth. Three different volumes, 5, 10, and 15 µL of each solvent [methanol (1), ethanol (4), hexane (3), and chloroform (2)], were tested. Kanamycin (km, 50 µg/mL) was used as a positive control. Pictures are representative of all experiments and tests conducted. The control illustrates the growth of strain 3016 onto TAP agar Petri dishes without solvents.

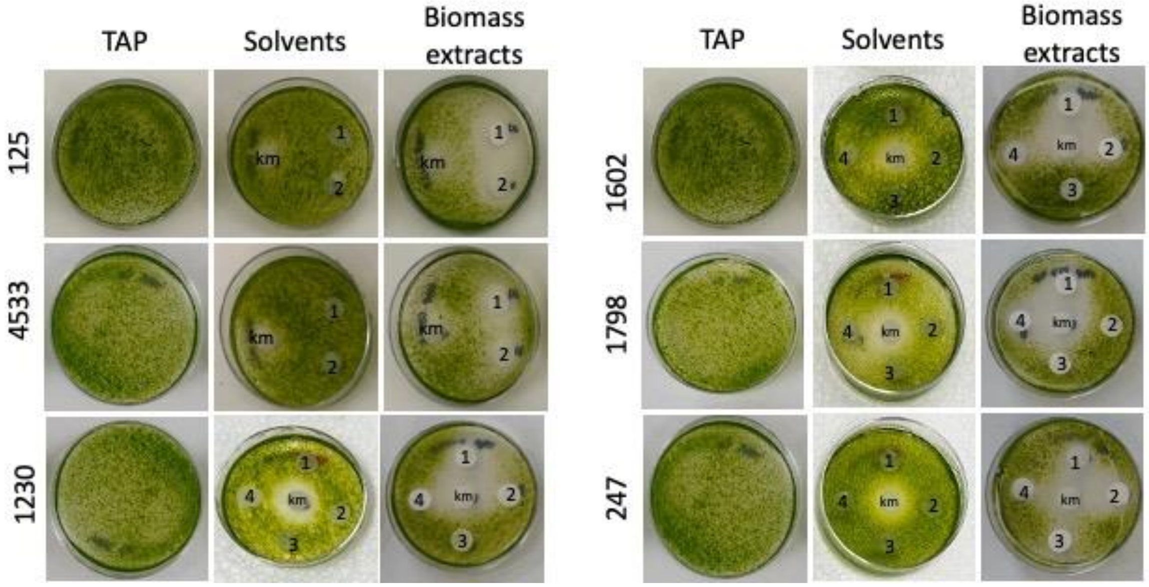

Figure S3. Biomass extracts of *Chlamydomonas*, *Chlorella*, and *Chloroidium* strains inhibit the growth of *C. sorokiniana* UTEX 3016. Disk-diffusion assay was conducted to evaluate the impact of biomass solvent extracts [methanol (1), ethanol (2), hexane (3), and chloroform (4)] produced from stationary-phase cultures of (a) *Chlamydomonas* strains (125 and 4533), (b) *Chlorella* strains (1602, 1798, and 1230), and *Chloroidium strain* (247) (see section 2). The strain *C. sorokiniana* 3016 was used as the target organism. Kanamycin (km, 50 µg/mL) was used as a positive control. The TAP agar Petri dishes show strain growth with no solvents or microalgae extracts. Independent experiments were repeated three times with three technical replicates.

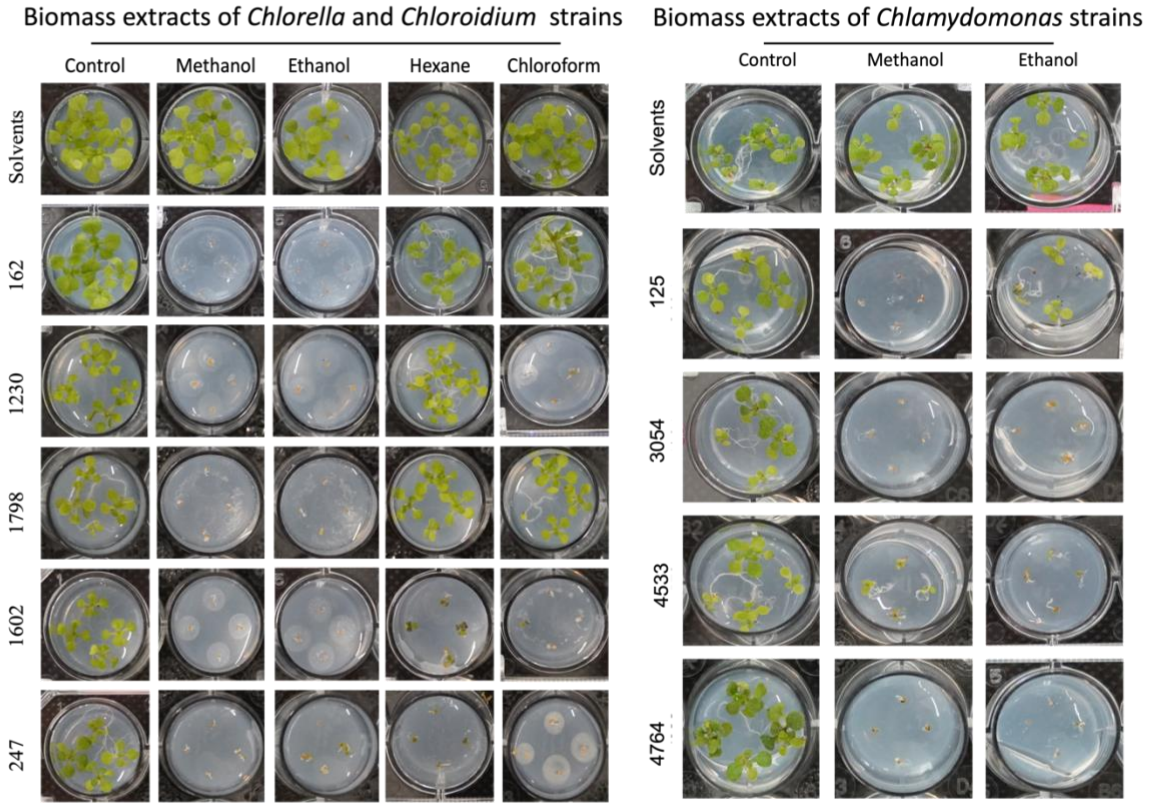

Figure S4. Biomass extracts of *Chlamydomonas*, *Chlorella*, and *Chloroidium* strains present herbicidal activity. Assessment of biomass extracts of *Chlorella* (162, 1230, 178, 1602), *Chloroidium* (247), and *Chlamydomonas* (3054, 4764, 125, 4533) strains for *Arabidopsis* growth and seed germination inhibition. Control conditions using MS growth medium with no microalgae extracts and solvents only were used to test seed germination. Please note that whitish spots onto the agar observed in some photographs are the residues of microalgae extracts. Photographs were taken 12 days after germination.

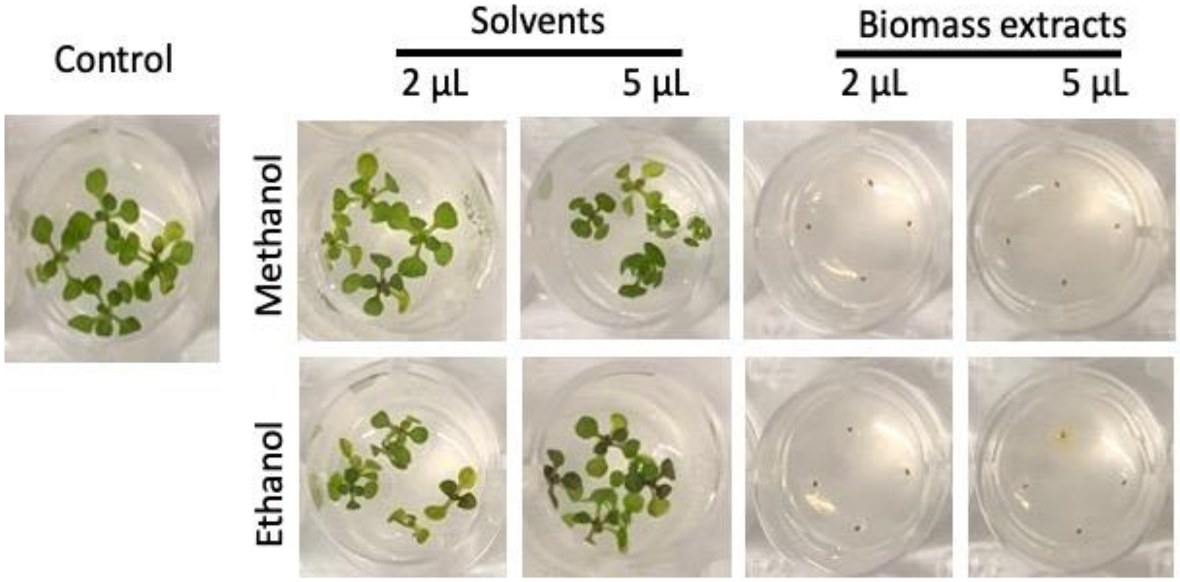

Figure S5. Assessment of biomass extracts of strain *C. reinhardtii* CC124 for *Arabidopsis* growth and seed germination inhibition with two volumes of the extract (2 µL and 5 µL). Control conditions using MS growth medium with no microalgae extracts and solvents only were used to test seed germination. Photographs were taken 18 days after germination.

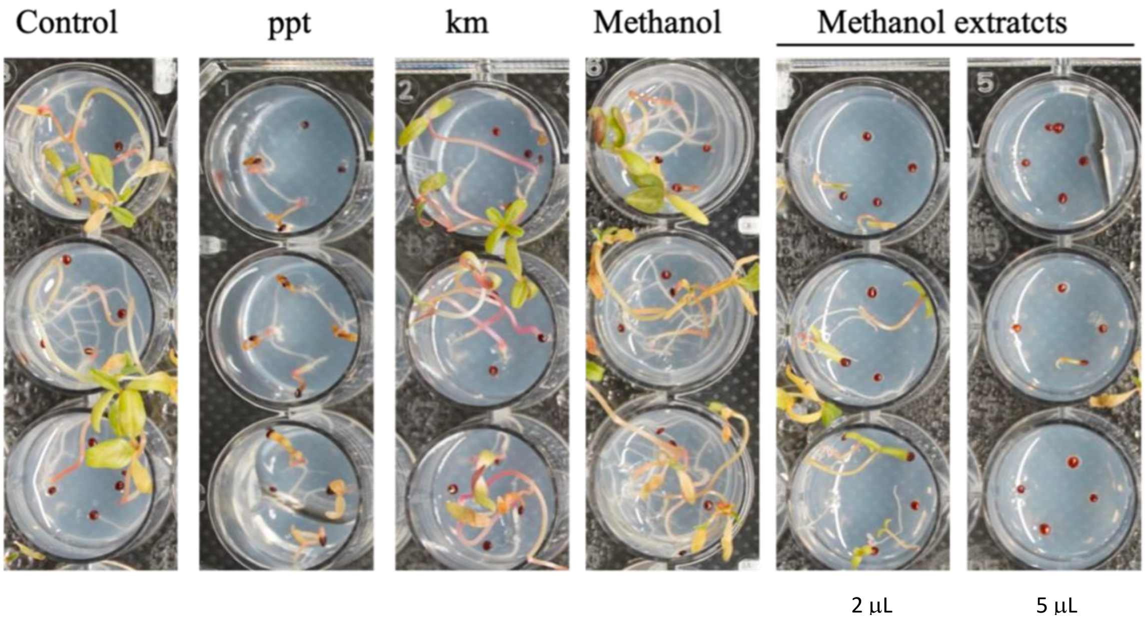

Figure S6. Methanol extracts of strain *C. reinhardtii* CC124 inhibits the growth of *A. palmeri*. Top view of 20-day-old *A. palmeri* seedlings treated with methanol biomass extracts of strain 124. Rows represent technical replicates of representative plants. Control indicates growth medium without solvent or biomass extract. Phosphinotricin (ppt, 15 μg/L) and kanamycin (km, 50 μg/mL) were used as positive controls. Volume indicates the volume of biomass extract utilized.

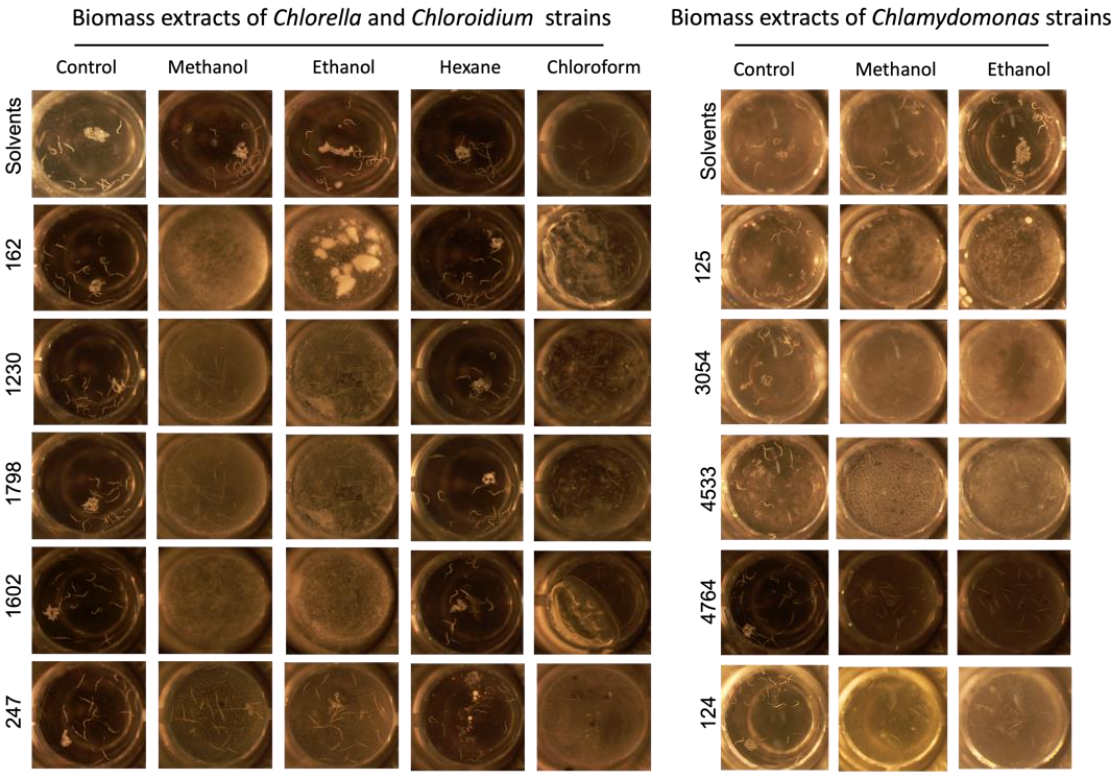

Figure S7. Biomass extracts of *Chlamydomonas*, *Chlorella* and *Chloroidium* strains present nematocidal activity. Assessment of the toxic effect of biomass extracts from *Chlorella* (162, 1230, 1798, 1602), *Chloroidium* (247), and *Chlamydomonas* (3054, 4764, 125, 4533, 124) strains on *C. elegans*. Living nematodes are visible as curved white strings, and dead nematodes appear as straight strings after 72 h of treatment. Control conditions with solvents only and no microalgae extracts were used to evaluate their effect on nematode viability.

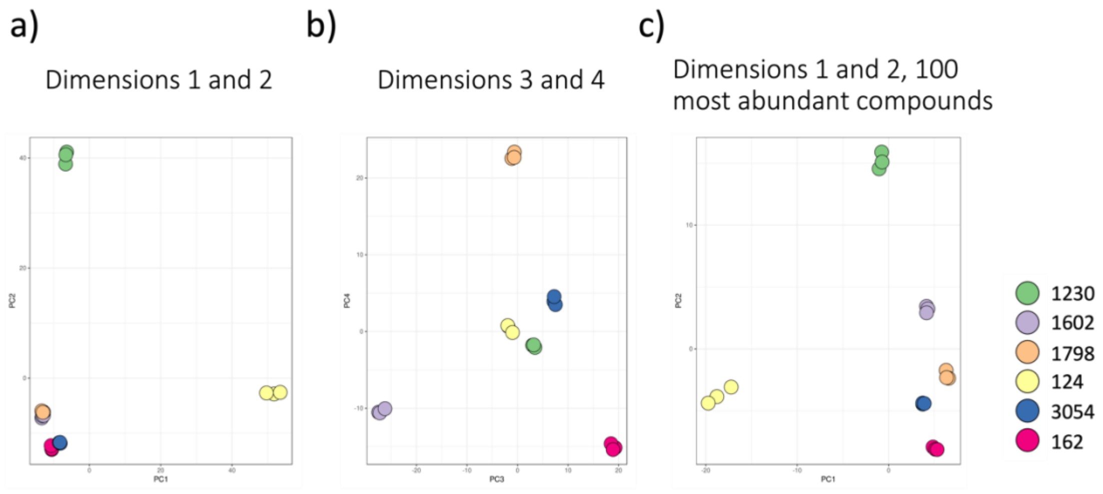

Figure S8. Principal Component Analysis of the named compounds **(a,b)** and the 100 more abundant metabolites **(c)** from the metabolite profiles analysis of strains 1230, 1602, 1798, 124, 3054, and 162 with Compound Discoverer. The normalized areas of named compounds were used for the analysis. Each dot represents a technical replicate.

Table S1. Pesticide activities of microalgae biomass extracts tested against *C. sorokiniana* UTEX 3016 (*Cs*), *A. thaliana* (*At*), and *C. elegans* (*Ce*).

	Solvent extraction											
	Methanol			Ethanol			Hexane			Chloroform		
**Target organism**	*Cs*	*At*	*Ce*	*Cs*	*At*	*Ce*	*Cs*	*At*	*Ce*	*Cs*	*At*	*Ce*
C. reinhardtii CC125	++	++	++	++	+	+	ND	ND	ND	ND	ND	0
C. reinhardtii CC124	++	++	++	++	++	++	ND	ND	ND	ND	ND	0
C. reinhardtii 4764	++	++	++	++	++	++	ND	ND	ND	ND	ND	0
C. reinhardtii CC4533	+	++	++	+	++	++	ND	ND	ND	ND	ND	0
C.reinhardtii CC3054	++	++	++	-	-	++	ND	ND	ND	ND	ND	0
Chlorella sp. EE162	++	++	++	-	++	++	-	-	-	-	-	0
Chlorella sp. 'anitrata' UTEX 1798	++	++	++	-	++	++	-	-	-	++	-	0
C. sorokiniana UTEX 1230	++	++	++	-	++	++	-	-	-	-	++	0
C. sorokiniana UTEX 1602	++	++	++	+	++	++	-	+	-	+	++	0
C. saccharophilum UTEX 247	+	+	++	++	+	++	+	+	-	-	++	0

For experiments with strain *C. sorokiniana* UTEX 3016 as the target organism, the halo of inhibition > 13 mm (++); the halo of inhibition < 13 mm (+); no activity (-); no data obtained (ND).
For experiments with *A. thaliana* as the target organism, germination inhibition (++); growth reduction (+); no activity (-); no data obtained (ND).
For experiments with *C. elegans* as the target organism, dead nematodes (++); reduction of nematode survival (+); no activity (-); no data obtained (ND); dead nematodes under control treatment with solvent only (0).

Table S2. *C. elegans* L1 larvae survival (%) after 72 h of treatment with microalgae biomass extracts.

	Solvents			
	Methanol	Ethanol	Hexane	Chloroform
Control	97	98	93	-
C. reinhardtii CC125	0	63	NA	-
C. reinhardtii CC124	0	0	NA	-
C. reinhardtii 4764	56	0	NA	-
C. reinhardtii CC3054	0	0	NA	-
C. reinhardtii CC4533	0	9	NA	-
Chlorella sp. EE162	0	0	94	-
Chlorella sp. 'anitrata' UTEX 1798	0	0	96	-
C. sorokiniana UTEX 1230	0	0	97	-
C. sorokiniana UTEX 1602	0	0	98	-
C. saccharophilum UTEX 247	8	4	95	-

The survival percentage of the nematodes was determined by counting the alive and dead nematodes under white light stimuli, in reference to the total nematodes treated. No data obtained (ND); dead nematodes under control treatment with solvent only (-).

Table S3. Major chemical classes of metabolites identified in methanol extracts of strain *C. sorokiniana* UTEX 1230.

	Group	Hits	Members
**Benzamides**	Benzamides	26	myo-Inositol; Coproporphyrin III; Pyrophaeophorbide a; 12-OPDA; Magnesium protoporphyrin; (S)-4-Amino-5-oxopentanoate; Magnesium protoporphyrin monomethyl ester; 13(S)-HPOT; (3Z)-Phytochromobilin; 4-(4-Deoxy-alpha-D-gluc-4-enuronosyl)-D-galacturonate; Echinenone; Gibberellin A12; 5-Hydroxyconiferaldehyde; Abscisic aldehyde; 22alpha-Hydroxy-campest-4-en-3-one; Adonixanthin; (22R,23R)-22,23-Dihydroxy-campest-4-en-3-one; 12-Oxo-9(Z)-dodecenoic acid; 9-Oxononanoic acid; 9,10-EOT; 10-Hydroxygeraniol; 10-Oxogeranial; Pheophorbide a; Red chlorophyll catabolite; Primary fluorescent chlorophyll catabolite; Caffeyl alcohol
**Amino acids and peptides**	Amino acids and peptides	19	Argininosuccinic acid; L-Glutamic acid; L-Phenylalanine; L-Alanine; L-Threonine; L-Isoleucine; L-Lysine; Sarcosine; Saccharopine; L-Arginine; L-Leucine; L-Homoserine; 4-Hydroxyproline; Citrulline; L-Glutamic gamma-semialdehyde; O-Acetylserine; 4-Guanidinobutanoic acid; L-Allothreonine; gamma-Glutamyl-beta-cyanoalanine
**Fatty Acids and Conjugates**	Fatty Acids and Conjugates	17	2-Ketobutyric acid; Acetoacetic acid; 5-Aminolevulinic acid; (S)-Methylmalonic acid semialdehyde; (S)-2-Acetolactate; Gamma-Aminobutyric acid; L-Alpha-aminobutyric acid; 2-Methyl-3-oxopropanoic acid; (R)-b-aminoisobutyric acid; Caprylic acid; Linoleic acid; Alpha-Linolenic acid; Docosahexaenoic acid; Arachidic acid; Gamma-Linolenic acid; Succinic acid; Methylmalonic acid
**Isoprenoids**	Isoprenoids	9	Farnesyl pyrophosphate; Zeaxanthin; Violaxanthin; Lutein; Perillyl aldehyde; Perillic acid; Phytyl diphosphate; Abscisic aldehyde; Gibberellin A12

**Sterols**	Sterols	8	Stigmasterol; 4,4-Dimethyl-5a-cholesta-8,24-dien-3-b-ol; Avenasterol; 4a-Methylfecosterol; 24-Methylenelophenol; 5-Dehydroepisterol; Delta7-Avenasterol; 3-Dehydroteasterone
**Eicosanoids**	Eicosanoids	7	Leukotriene B4; 5(S)-Hydroperoxyeicosatetraenoic acid; Prostaglandin E2; Prostaglandin H2; 15(S)-HETE; 15(S)-HPETE; 5-HETE
**Purines**	Purines	5	Adenine; Adenosine monophosphate; Adenosine; Deoxyguanosine; Guanosine monophosphate
**Monosaccharides**	Monosaccharides	5	Glycerol; N-Acetyl-D-glucosamine; L-Galactose; D-Glucose; D-Mannose
**Sphingoid bases**	Sphingoid bases	4	Sphinganine; 3-Dehydrosphinganine; Phytosphingosine; Sphingosine
**Prenol lipids**	Prenol lipids	4	Neoxanthin; epsilon-Tocopherol; Gibberellin A12 7-aldehyde; Menaquinol
**Pyrimidines**	Pyrimidines	3	Cytidine monophosphate; Thymidine; Uridine 5'-monophosphate
**Octadecanoids**	Octadecanoids	3	Jasmonic acid; 9,10-Epoxystearic acid; Methyl jasmonate
**TCA acids**	TCA acids	2	Fumaric acid; Succinic acid
**Benzoic acids**	Benzoic acids	2	2-Aminobenzoic acid; p-Aminobenzoic acid
**Cinnamic acids**	Cinnamic acids	2	trans-Ferulic acid; Caffeate
This table summarizes the matched metabolite sets ranked by their *P* values.			

**Table S4.** Major chemical classes of metabolites identified in methanol extracts of strain *C. sorokiniana* UTEX 1602.			
	**Group**	**Hits**	**Members**
**Benzamides**	Benzamides	30	myo-Inositol; Coproporphyrin III; Protochlorophyllide; Tropine; 12-OPDA; Magnesium protoporphyrin; Fecosterol; 13(S)-HPOT; (3Z)-Phytochromobilin; Antheraxanthin; Echinenone; 2-C-Methyl-D-erythritol 4-phosphate; 13(1)-Oxo-magnesium-protoporphyrin IX 13-monomethyl ester; Divinyl chlorophyllide a; 5-Hydroxyconiferaldehyde; Abscisic aldehyde; 22alpha-Hydroxy-campest-4-en-3-one; 3'-Hydroxyechinenone; 3-Hydroxyechinenone; Adonixanthin; (22R,23R)-22,23-Dihydroxy-campest-4-en-3-one; 12-Oxo-9(Z)-dodecenoic acid; 9-Oxononanoic acid; 9,10-EOT; 10-Hydroxygeraniol; 10-Oxogeranial; Pheophorbide a; Red chlorophyll catabolite; Primary fluorescent chlorophyll catabolite; Caffeyl alcohol
**Fatty Acids and Conjugates**	Fatty Acids and Conjugates	17	2-Hydroxybutyric acid; (S)-3-Hydroxyisobutyric acid; (S)-2-Acetolactate; Alpha-ketoisovaleric acid; Gamma-Aminobutyric acid; L-Alpha-aminobutyric acid; Aminoadipic acid; Diaminopimelic acid; (R)-b-aminoisobutyric acid; Linoleic acid; Arachidonic acid; Alpha-Linolenic acid; Arachidic acid; 8,11,14-Eicosatrienoic acid; Gamma-Linolenic acid; Succinic acid; Methylmalonic acid
**Amino acids and peptides**	Amino acids and peptides	15	Argininosuccinic acid; L-Glutamic acid; L-Alanine; L-Proline; L-Threonine; L-Isoleucine; L-Lysine; Sarcosine; Saccharopine; L-Leucine; L-Homoserine; O-Acetylserine; 4-Guanidinobutanoic acid; L-Allothreonine; gamma-Glutamyl-beta-cyanoalanine
**Sterols**	Sterols	13	7-Dehydrocholesterol; Cholesterol; Lathosterol; Desmosterol; Zymosterol intermediate 2; 5a-Cholest-8-en-3b-ol; 5-Dehydroepisterol; Brassicasterol; Campest-4-

			en-3-one; Episterol; Fecosterol; Obtusifoliol; 3-Dehydroteasterone
**Isoprenoids**	Isoprenoids	7	Zeaxanthin; Violaxanthin; Lutein; Perillyl aldehyde; Perillic acid; Abscisic aldehyde; Antheraxanthin
**Purines**	Purines	5	Adenine; Adenosine monophosphate; Adenosine; Deoxyguanosine; Xanthine
**Monosaccharides**	Monosaccharides	5	Glycerol; N-Acetyl-D-glucosamine; L-Galactose; D-Glucose; D-Mannose
**Eicosanoids**	Eicosanoids	5	Leukotriene B4; 5(S)-Hydroperoxyeicosatetraenoic acid; 15(S)-HETE; 15(S)-HPETE; 5-HETE
**Steroids**	Steroids	5	Obtusifoliol; Episterol; 24-Methylenecholesterol; Campest-4-en-3-one; 3-Dehydroteasterone
**Sphingoid bases**	Sphingoid bases	4	Sphinganine; 3-Dehydrosphinganine; Phytosphingosine; Sphingosine
**Pyrimidines**	Pyrimidines	3	Thymidine; Uridine; Uracil
**TCA acids**	TCA acids	3	Citric acid; Fumaric acid; Succinic acid
**Cinnamic acids**	Cinnamic acids	3	trans-Ferulic acid; Sinapic acid; Caffeate
**Prenol lipids**	Prenol lipids	3	Neoxanthin; epsilon-Tocopherol; Menaquinol
**Disaccharides**	Disaccharides	2	Sucrose; Trehalose
This table summarizes the matched metabolite sets ranked by their *P* values.			

**Table S5.** Major chemical classes of metabolites identified in methanol extracts of strain *Chlorella* sp. 'anitrata' 1798.			
	**Group**	**Hits**	**Members**
**Benzamides**	Benzamides	27	myo-Inositol; Coproporphyrin III; Pyrophaeophorbide a; 12-OPDA; 13(S)-HPOT; (1R,6R)-6-Hydroxy-2-succinylcyclohexa-2,4-diene-1-carboxylate; (3Z)-Phytochromobilin; Antheraxanthin; Echinenone; 2-C-Methyl-D-erythritol 4-phosphate; ent-7alpha-Hydroxykaur-16-en-19-oic acid; 5-Hydroxyconiferaldehyde; Cathasterone; 22alpha-Hydroxy-campest-4-en-3-one; 3-Dehydro-6-deoxoteasterone; 3'-Hydroxyechinenone; 3-Hydroxyechinenone; Adonixanthin; (22R,23R)-22,23-Dihydroxycampesterol; (22R,23R)-22,23-Dihydroxy-campest-4-en-3-one; 12-Oxo-9(Z)-dodecenoic acid; 9-Oxononanoic acid; 9,10-EOT; Pheophorbide a; Red chlorophyll catabolite; Primary fluorescent chlorophyll catabolite; Caffeyl alcohol
**Fatty Acids and Conjugates**	Fatty Acids and Conjugates	21	2-Ketobutyric acid; 2-Hydroxybutyric acid; (S)-3-Hydroxyisobutyric acid; Acetoacetic acid; Propionic acid; Hydroxypropionic acid; (S)-Methylmalonic acid semialdehyde; (S)-2-Acetolactate; Gamma-Aminobutyric acid; L-Alpha-aminobutyric acid; Aminoadipic acid; 2-Methyl-3-oxopropanoic acid; Diaminopimelic acid; (R)-b-aminoisobutyric acid; Linoleic acid; Arachidonic acid; Alpha-Linolenic acid; Arachidic acid; Gamma-Linolenic acid; Succinic acid; Methylmalonic acid
**Amino acids and peptides**	Amino acids and peptides	15	L-Glutamic acid; L-Phenylalanine; L-Alanine; L-Proline; L-Threonine; L-Isoleucine; L-Histidine; Sarcosine; L-Arginine; L-Leucine; L-Homoserine; O-Acetylserine; 4-Acetamidobutanoic acid; L-Allothreonine; gamma-Glutamyl-beta-cyanoalanine
**Isoprenoids**	Isoprenoids	6	Zeaxanthin; Violaxanthin; Lutein; Perillyl aldehyde; Phytyl diphosphate; Antheraxanthin

**Monosaccharides**	Monosaccharides	5	Glycerol; Dihydroxyacetone; L-Galactose; D-Glucose; D-Mannose
**Prenol lipids**	Prenol lipids	5	Neoxanthin; epsilon-Tocopherol; (ent-6alpha,7alpha)-6,7-Dihydroxy-16-kauren-19-oic acid; Gibberellin A12 7-aldehyde; Menaquinol
**Purines**	Purines	4	Adenine; Adenosine monophosphate; Adenosine; Deoxyguanosine
**Sphingoid bases**	Sphingoid bases	4	Sphinganine; 3-Dehydrosphinganine; Phytosphingosine; Sphingosine
**Quinones and hydroquinones**	Quinones and hydroquinones	3	Alpha-Tocotrienol; Gamma-Tocotrienol; Vitamin K2
**Sterols**	Sterols	3	5-Dehydroepisterol; Cathasterone; 3-Dehydro-6-deoxoteasterone
**Disaccharides**	Disaccharides	2	Sucrose; Trehalose
**Benzoic acids**	Benzoic acids	2	2-Aminobenzoic acid; p-Aminobenzoic acid
**Cinnamic acids**	Cinnamic acids	2	trans-Ferulic acid; Sinapic acid
**Porphyrins**	Porphyrins	2	Coproporphyrinogen III; Coproporphyrinogen I
**Octadecanoids**	Octadecanoids	2	9,10-Epoxystearic acid; Methyl jasmonate

This table summarizes the matched metabolite sets ranked by their *P* values.

**Table S6.** Major chemical classes of metabolites identified in methanol extracts of strain *C. reinhardtii* CC124.			
	**Group**	**Hits**	**Members**
**Benzamides**	Benzamides	27	Coproporphyrin III; Pyrophaeophorbide a; Tropinone; 12-OPDA; Magnesium protoporphyrin monomethyl ester; 13(S)-HPOT; (3Z)-Phytochromobilin; Echinenone; Gibberellin A12; ent-7alpha-Hydroxykaur-16-en-19-oic acid; 5-Hydroxyconiferaldehyde; Abscisic aldehyde; Castasterone; 22alpha-Hydroxy-campest-4-en-3-one; Adonixanthin; (22R,23R)-22,23-Dihydroxy-campest-4-en-3-one; 12-Oxo-9(Z)-dodecenoic acid; 9-Oxononanoic acid; 9,10-EOT; 10-Hydroxygeraniol; 10-Oxogeranial; 7-Oxateasterone; 7-Oxatyphasterol; Pheophorbide a; Red chlorophyll catabolite; Primary fluorescent chlorophyll catabolite; (GlcN)1 (Ino(acyl)-P)1 (Man)3 (EtN)1 (P)1
**Fatty Acids and Conjugates**	Fatty Acids and Conjugates	15	(S)-2-Acetolactate; Gamma-Aminobutyric acid; L-Alpha-aminobutyric acid; (R)-b-aminoisobutyric acid; Palmitic acid; Caprylic acid; Capric acid; Linoleic acid; Stearic acid; Alpha-Linolenic acid; Docosahexaenoic acid; Arachidic acid; Gamma-Linolenic acid; Succinic acid; Methylmalonic acid
**Isoprenoids**	Isoprenoids	10	2-trans,6-trans-Farnesal; Farnesyl pyrophosphate; Zeaxanthin; Violaxanthin; Lutein; Perillyl aldehyde; Perillic acid; (S)-Abscisic acid; Abscisic aldehyde; Gibberellin A12
**Amino acids and peptides**	Amino acids and peptides	8	L-Tyrosine; L-Phenylalanine; L-Isoleucine; L-Lysine; Saccharopine; L-Leucine; 4-Acetamidobutanoic acid; N-Acetyl-L-glutamate 5-semialdehyde
**Prenol lipids**	Prenol lipids	5	Neoxanthin; epsilon-Tocopherol; (ent-6alpha,7alpha)-6,7-Dihydroxy-16-kauren-19-oic acid; Gibberellin A12 7-aldehyde; Menaquinol
**Purines**	Purines	4	Adenosine monophosphate; Adenosine; Deoxyguanosine; Deoxyadenosine
**Sphingoid bases**	Sphingoid bases	4	Sphinganine; 3-Dehydrosphinganine; Phytosphingosine; Sphingosine
**Eicosanoids**	Eicosanoids	3	Leukotriene A4; 15(S)-HETE; 5-HETE

**Quinones and hydroquinones**	Quinones and hydroquinones	3	Alpha-Tocotrienol; Gamma-Tocotrienol; Vitamin K2
**Sterols**	Sterols	3	5-Dehydroepisterol; Castasterone; 3-Dehydroteasterone
**TCA acids**	TCA acids	2	Fumaric acid; Succinic acid
**Benzoic acids**	Benzoic acids	2	2-Aminobenzoic acid; p-Aminobenzoic acid
**Benzenes**	Benzenes	2	4-Hydroxyphenylpyruvic acid; Phenylethylamine
**Cinnamic acids**	Cinnamic acids	2	trans-Ferulic acid; Caffeate
**Porphyrins**	Porphyrins	2	Coproporphyrinogen III; Coproporphyrinogen I
This table summarizes the matched metabolite sets ranked by their *P* values.			

Table S7. Major chemical classes of metabolites identified in methanol extracts of strain *C. reinhardtii* CC3054.

	Group	Hits	Members
**Benzamides**	Benzamides	28	myo-Inositol; Coproporphyrin III; Gibberellin A1; 12-OPDA; 13(S)-HPOT; (3Z)-Phytochromobilin; Gibberellin A29; Antheraxanthin; Echinenone; 2-C-Methyl-D-erythritol 4-phosphate; Gibberellin A12; Gibberellin A34; 5-Hydroxyconiferaldehyde; Abscisic aldehyde; Castasterone; 22alpha-Hydroxy-campest-4-en-3-one; 3'-Hydroxyechinenone; 3-Hydroxyechinenone; Adonixanthin; 12-Oxo-9(Z)-dodecenoic acid; 9-Oxononanoic acid; 9,10-EOT; 10-Hydroxygeraniol; 10-Oxogeranial; 7-Oxateasterone; 7-Oxatyphasterol; Red chlorophyll catabolite; Caffeyl alcohol
**Amino acids and peptides**	Amino acids and peptides	11	L-Phenylalanine; L-Alanine; L-Threonine; L-Isoleucine; Sarcosine; Saccharopine; L-Leucine; L-Homoserine; 4-Acetamidobutanoic acid; L-Allothreonine; gamma-Glutamyl-beta-cyanoalanine
**Isoprenoids**	Isoprenoids	11	Zeaxanthin; Violaxanthin; Lutein; Perillyl aldehyde; Perillic acid; Phytyl diphosphate; Abscisic aldehyde; Antheraxanthin; Gibberellin A1; Gibberellin A12; Gibberellin A34
**Fatty Acids and Conjugates**	Fatty Acids and Conjugates	8	(S)-2-Acetolactate; Linoleic acid; Arachidonic acid; Alpha-Linolenic acid; Gamma-Linolenic acid; Dethiobiotin; Succinic acid; Methylmalonic acid
**Prenol lipids**	Prenol lipids	5	Neoxanthin; epsilon-Tocopherol; (ent-6alpha,7alpha)-6,7-Dihydroxy-16-kauren-19-oic acid; Gibberellin A12 7-aldehyde; Menaquinol
**Sphingoid bases**	Sphingoid bases	4	Sphinganine; 3-Dehydrosphinganine; Phytosphingosine; Sphingosine
**Purines**	Purines	3	Adenosine monophosphate; Adenosine; Deoxyguanosine
**Monosaccharides**	Monosaccharides	3	L-Galactose; D-Glucose; D-Mannose

**Sterols**	Sterols	3	5-Dehydroepisterol; Castasterone; 3-Dehydroteasterone
**Disaccharides**	Disaccharides	2	Sucrose; Trehalose
**Benzoic acids**	Benzoic acids	2	2-Aminobenzoic acid; p-Aminobenzoic acid
**Cinnamic acids**	Cinnamic acids	2	trans-Ferulic acid; Caffeate
**Porphyrins**	Porphyrins	2	Coproporphyrinogen III; Coproporphyrinogen I
**Organooxygen compounds**	Organooxygen compounds	2	N4-Acetylaminobutanal; Beta-Cortol
**Aldehydes**	Aldehydes	2	3-Aminopropionaldehyde; 1-beta-D-Glucopyranosyl-4-D-glucopyranose
This table summarizes the matched metabolite sets ranked by their *P* values.			

**Table S8.** Major chemical classes of metabolites identified in methanol extracts of strain *Chlorella* sp. UTEX EE162.			
	**Group**	**Hits**	**Members**
**Benzamides**	Benzamides	19	Coproporphyrin III; Pyrophaeophorbide a; 12-OPDA; 13(S)-HPOT; (3Z)-Phytochromobilin; Antheraxanthin; Echinenone; 5-Hydroxyconiferaldehyde; Abscisic aldehyde; 3'-Hydroxyechinenone; 3-Hydroxyechinenone; Adonixanthin; 12-Oxo-9(Z)-dodecenoic acid; 9-Oxononanoic acid; 9,10-EOT; 10-Hydroxygeraniol; 10-Oxogeranial; Red chlorophyll catabolite; Caffeyl alcohol
**Amino acids and peptides**	Amino acids and peptides	15	L-Glutamic acid; L-Phenylalanine; L-Alanine; L-Threonine; L-Isoleucine; L-Histidine; L-Lysine; Sarcosine; L-Arginine; L-Leucine; L-Homoserine; O-Acetylserine; 4-Acetamidobutanoic acid; L-Allothreonine; Ophthalmic acid
**Fatty Acids and Conjugates**	Fatty Acids and Conjugates	13	2-Ketobutyric acid; Acetoacetic acid; (S)-Methylmalonic acid semialdehyde; (S)-2-Acetolactate; Aminoadipic acid; 2-Methyl-3-oxopropanoic acid; Oleic acid; Linoleic acid; Alpha-Linolenic acid; Arachidic acid; Gamma-Linolenic acid; Succinic acid; Methylmalonic acid
**Isoprenoids**	Isoprenoids	9	2-trans,6-trans-Farnesal; Zeaxanthin; Violaxanthin; Canthaxanthin; Lutein; Perillyl aldehyde; Perillic acid; Abscisic aldehyde; Antheraxanthin
**Sterols**	Sterols	9	Cholesterol; Stigmasterol; Lathosterol; 4,4-Dimethyl-5a-cholesta-8,24-dien-3-b-ol; Avenasterol; 5a-Cholest-8-en-3b-ol; 4a-Methylfecosterol; 24-Methylenelophenol; Delta7-Avenasterol
**Sphingoid bases**	Sphingoid bases	4	Sphinganine; 3-Dehydrosphinganine; Phytosphingosine; Sphingosine
**Purines**	Purines	3	Adenosine monophosphate; Adenosine; Deoxyguanosine
**Cinnamic acids**	Cinnamic acids	3	trans-Ferulic acid; Sinapic acid; Caffeate
**Disaccharides**	Disaccharides	2	Sucrose; Trehalose
**Benzoic acids**	Benzoic acids	2	2-Aminobenzoic acid; p-Aminobenzoic acid
**Benzenes**	Benzenes	2	Tyramine; 4-Hydroxyphenylpyruvic acid

**Octadecanoids**	Octadecanoids	2	9,10-Epoxystearic acid; Methyl jasmonate
**Fatty Acyls**	Fatty Acyls	2	9(S)-HPODE; Methyl jasmonate
**Pyrimidines**	Pyrimidines	1	Cytidine monophosphate
**Pyridines**	Pyridines	1	Nicotinic acid
This table summarizes the matched metabolite sets ranked by their *P* values.			
